# Associations between DNA methylation and gene regulation depend on chromatin accessibility during transgenerational plasticity

**DOI:** 10.1186/s12915-023-01645-8

**Published:** 2023-06-26

**Authors:** Samuel N. Bogan, Marie E. Strader, Gretchen E. Hofmann

**Affiliations:** 1 Department of Ecology, Evolution and Marine Biology, University of California Santa Barbara, Santa Barbara, USA; 2 Department of Biology, Texas A&M University, College Station, USA

**Keywords:** Epigenetics, DNA Methylation, Gene expression, Environment, Invertebrate, Chromatin

## Abstract

**Background:**

Epigenetic processes are proposed to be a mechanism regulating gene expression during phenotypic plasticity. However, environmentally induced changes in DNA methylation exhibit little-to-no association with differential gene expression in metazoans at a transcriptome-wide level. It remains unexplored whether associations between environmentally induced differential methylation and expression are contingent upon other epigenomic processes such as chromatin accessibility. We quantified methylation and gene expression in larvae of the purple sea urchin *Strongylocentrotus purpuratus* exposed to different ecologically relevant conditions during gametogenesis (maternal conditioning) and modeled changes in gene expression and splicing resulting from maternal conditioning as functions of differential methylation, incorporating covariates for genomic features and chromatin accessibility. We detected significant interactions between differential methylation, chromatin accessibility, and genic feature type associated with differential expression and splicing.

**Results:**

Differential gene body methylation had significantly stronger effects on expression among genes with poorly accessible transcriptional start sites while baseline transcript abundance influenced the direction of this effect. Transcriptional responses to maternal conditioning were 4–13 × more likely when accounting for interactions between methylation and chromatin accessibility, demonstrating that the relationship between differential methylation and gene regulation is partially explained by chromatin state.

**Conclusions:**

DNA methylation likely possesses multiple associations with gene regulation during transgenerational plasticity in *S. purpuratus* and potentially other metazoans*,* but its effects are dependent on chromatin accessibility and underlying genic features.

**Supplementary Information:**

The online version contains supplementary material available at 10.1186/s12915-023-01645-8.

## Background

Epigenetic modifications to the genome such as DNA methylation are one suite of regulatory factors that, in some cases, underpin phenotypic plasticity by affecting changes in transcription [1] and subsequent phenotypes [2]. In particular, epigenetic variation is hypothesized to drive transgenerational plasticity (TGP): the influence of parental environment on the phenotype of offspring and latter generations via non-genetic means [3–5]. Connections between DNA methylation, plasticity, and acclimatization hinge on how and whether DNA methylation influences gene regulation. At a whole genome level, invertebrate DNA methylation exhibits negligible relationships with differential expression (DE) and other modes of gene regulation in environmental studies [6]. These poor associations are frequently observed in non-mammalian vertebrates as well [7–13], calling into question an epigenetic basis for acclimatization among many metazoans. However, DNA methylation is unlikely to influence gene expression independent of other epigenetic and genetic factors [14]. Using the purple sea urchin *Strongylocentrotus purpuratus* as a model invertebrate, we tested the hypothesis that the effects of differential methylation (DM) on gene regulation (differential expression and alternative splicing) are contingent upon additional epigenomic and genomic states such as chromatin accessibility and genic architecture (the frequency, length, and arrangement of features such as promoters, exons, and introns) by integrating larval RNA-seq and reduced representation bisulfite sequencing (RRBS) data from our study of transgenerational plasticity [15] with publicly available ATAC-seq data for *S. purpuratus* larvae.

Multiple lines of evidence support DNA methylation’s association with plasticity in response to changing environments, but direct or indirect causal mechanisms remain unknown. Temporospatial environmental variation has been linked to modifications in invertebrate methylomes independent of genetic variation, demonstrating a potential role for DM during acclimatization [16–18]. DM of genes, gene modules, or whole genomes induced by environmental variation are associated with performance traits in stony corals, mollusks, crustaceans, and insects [18–21]. Causative tests of DNA methylation’s effect on phenotype conducted in vertebrates, arthropods, and cnidarians have demonstrated significant effects of DNA methyltransferase inhibition on changes in performance, development, and survival across temperature [22–25]. By contrast, inducible changes in invertebrate gene expression in response to environmental variation have frequently possessed insignificant relationships with differential gene body methylation or GBM at a transcriptome-wide level [6, 15, 20, 26, 27]. This lack of relationship between transcriptome-wide DM and DE is also evident across environmental studies of non-mammalian vertebrates to date including fishes [7–10], birds [12], and reptiles [13]. If environmental variation in DNA methylation is associated with or influences phenotype in some invertebrates and metazoans, how does this occur in the absence of an association with gene expression?

DNA methylation’s effects on gene regulation and its interactions with other epigenetic factors can both be numerous. Most invertebrate phyla exhibit sparsely methylated genomes punctuated by high levels of CpG methylation within gene bodies [28–31]. GBM positively correlates with baseline gene expression in cnidarians [6, 19, 29], bivalve mollusks of *Crassostrea sp*. [26, 27], arthropods [31–33], and vertebrates [34]. However, experimental demethylation of gene bodies in plants [35] and whole genomes in invertebrates [36] has resulted in upregulation and downregulation of corresponding genes, calling into question whether this association is obligately positive. Alternative splicing can be an important molecular response to environmental variation [37, 38] and is associated with baseline GBM in some invertebrates [31–35]. Here, baseline GBM refers to the percent methylation of a gene controlling against environmental effects: if differential GBM can be represented by a slope across environments, baseline GBM is that slope’s intercept. Thus far, changes in alternative splicing in response to environmental variation have shown weak relationships with differential GBM [12]. Invertebrate DNA methylation is also associated with chromatin state [31, 39] and the suppression of spurious intragenic transcription [19]. For example, DE between cell types is more strongly associated with DM when accompanied by differential chromatin accessibility [40]. Relationships between DM and gene expression are also dependent on genic architecture such that enhanced or silenced expression can be driven by changes to DNA methylation at specific genic features such as promoters and/or exons while variation in methylation at other genic regions can yield lesser effects [33, 34, 41]. Determining the function of DM during transcriptional responses to the environment thus requires an integrated approach that considers genic architecture, additional epigenetic states such as chromatin accessibility, and multiple modes of gene regulation. Such interactions between chromatin state and DNA methylation remain untested in the context of environmental adaptation and acclimation.

The purple sea urchin *Strongylocentrotus purpuratus* is a uniquely poised model invertebrate in which to conduct an integrative test of DNA methylation’s regulatory roles during phenotypic plasticity. *S. purpuratus* is an abundant herbivore distributed throughout North America’s Pacific subtidal kelp forests and rocky intertidal [42]. Populations inhabiting environmental gradients or mosaics exhibit genetic evidence of local adaptation and interpopulation variation in performance and gene expression under ecologically relevant stress [43–45]. TGP linked to maternal effects has been observed in *S. purpuratus* for traits including egg protein content, larval body size, gene expression, and DNA methylation [15, 46–50]. Maternal conditioning of *S. purpuratus* to abiotic conditions mimicking coastal upwelling can induce 3–6 × greater DM in offspring larvae relative to the effects of larval development under upwelling [15, 50]. These results suggest a function for DM in facilitating TGP’s effects on gene expression, but negligible overlap between DM CpGs and DE genes has left that role ambiguous [15]. Accounting for interactions between DNA methylation and chromatin accessibility in *S. purpuratus*, which may better explain epigenetic effects on transcription, is made possible by the species’ use as a model of deuterostome embryology, yielding developmental time series of ATAC-seq spanning the two-cell embryo to late prism larvae.

To elucidate the gene regulatory roles of DM during TGP, we quantified changes in DNA methylation, gene expression, and alternative splicing in prism larvae of *S. purpuratus* induced by maternal exposure to experimental upwelling during gametogenesis, an ecologically relevant, abiotic stress. Coastal upwelling increases *p*CO_2_ and decreases temperature as a result of wind-driven upward movement of deep, cold seawater [51]. We then modeled DE and splicing as functions of DM, genic feature type, and chromatin accessibility to test the hypothesis that DNA methylation’s regulatory role is contingent upon genic architecture and chromatin accessibility. To pursue these aims, we integrated data from Strader et al., initially exhibiting limited overlap between DM and DE genes and ATAC-seq measures of chromatin accessibility during the *S. purpuratus* prism stage [15, 52–54] and applied a Bayesian workflow that fitted multiple model iterations spanning low–to–high dimensionality before selecting the most likely model and evaluating effects of its parameters averaged across all iterations. Model selection and parameter evaluation was facilitated by Bayes factor tests comparing marginal likelihoods: the probability of a model or effect given one’s data. Bayes factor tests have generally been shown to reduce error in model predictions compared to other model selection methods, improving false positive predictions and reproducibility [55–58].

## Results

**Fig. 1:**
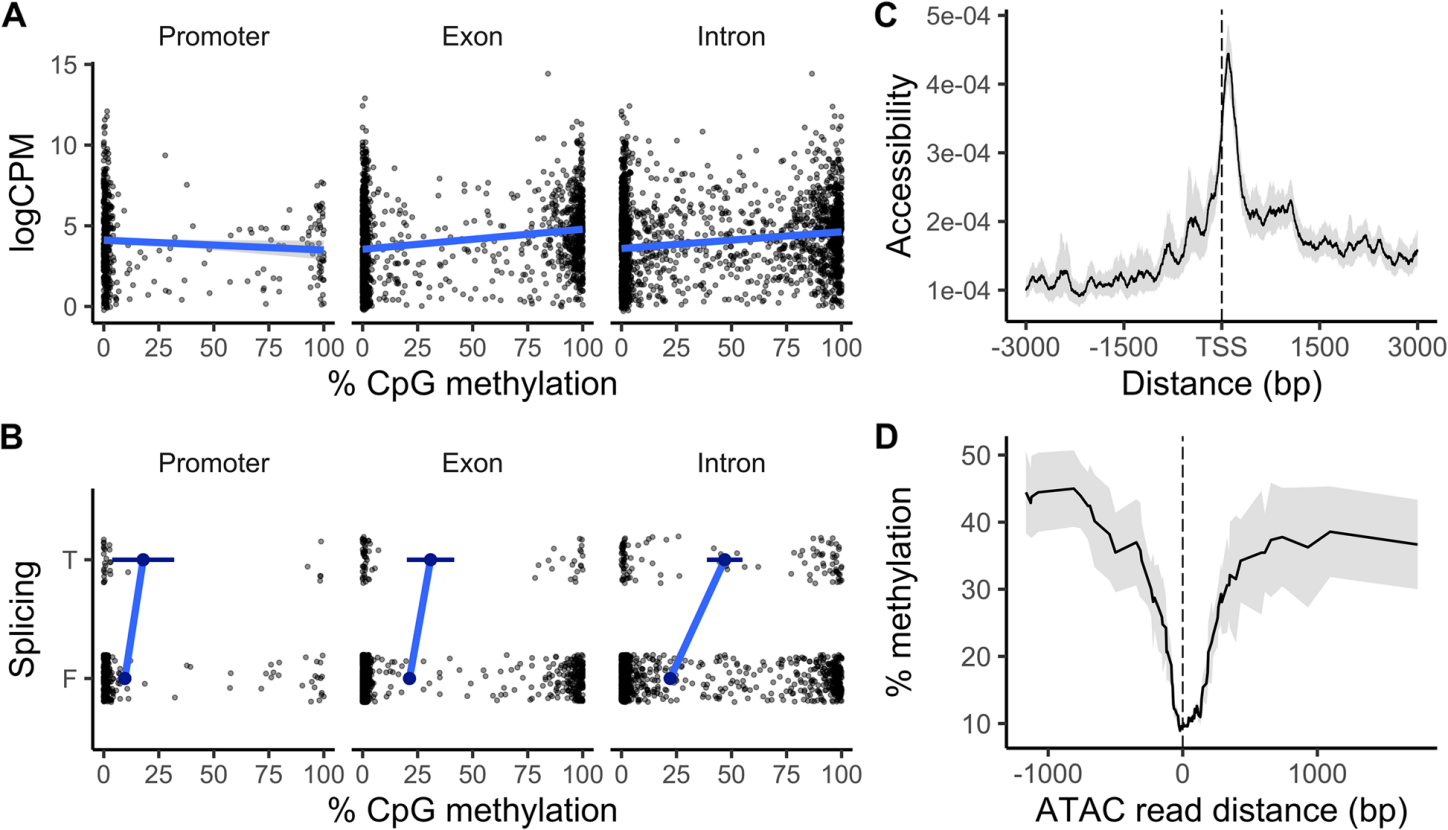
Relationships between constitutive DNA methylation, expression, and chromatin accessibility in larval *Strongylocentrotus purpuratus*. **A** Median percent methylation averaged across CpGs in the promoter, introns, and exons per gene is plotted against transcript abundance measured as logCPM. Each point depicts a single gene. **B** A binary value representing whether or not a gene was found to have transcript variants is plotted across averaged median methylation of genes’ promoters, introns, and exons. Small black points depict single genes. Large blue points depict average DNA methylation among genes with or without transcript variants ± 95% CI. T/F values are spread across the *y*-axis to reduce overlap among points and better visualize their distribution across CpG methylation. **C** A loess trend of mean ATAC-seq read density (i.e., chromatin accessibility) ± 95% CI is plotted across distance to transcriptional start sites (TSS). **D** A 50-bp sliding window average of % CpG methylation ± 95% CI is plotted across distance to accessible chromatin regions for which all ATAC-seq replicates shared consensus for accessibility. *n* = 12 RNA-seq and RRBS replicate libraries; *n* = 3 ATAC-seq replicate libraries

**Fig. 2:**
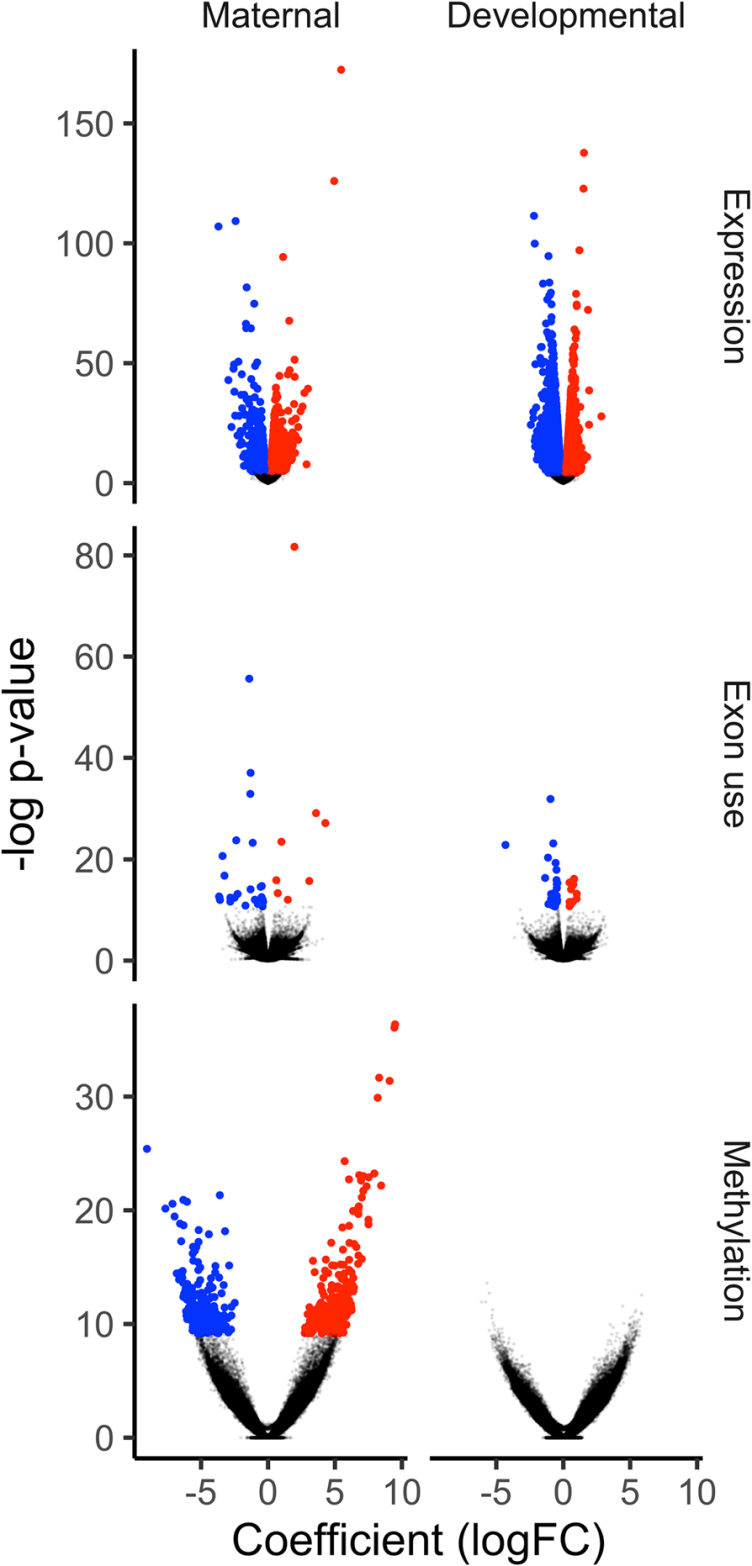
Molecular responses to developmental and maternal upwelling exposure. Volcano plots depicting differential expression (DE), differential exon use (DEU), and differential methylation (DM). DE is depicted by measures of genewise -log_2_*p*-values and log_2_FC. DEU (e.g., splicing) is depicted as exon-level -log_2_*p*-values, and ∆logFC coefficients. DM is depicted as CpG-level -log_2_*p*-values and log_2_FC of methylation. Red and blue points depict significant positive and negative coefficients, respectively (FDR < 0.05). *n* = 12 RNA-seq and RRBS replicate libraries

**Fig. 3:**
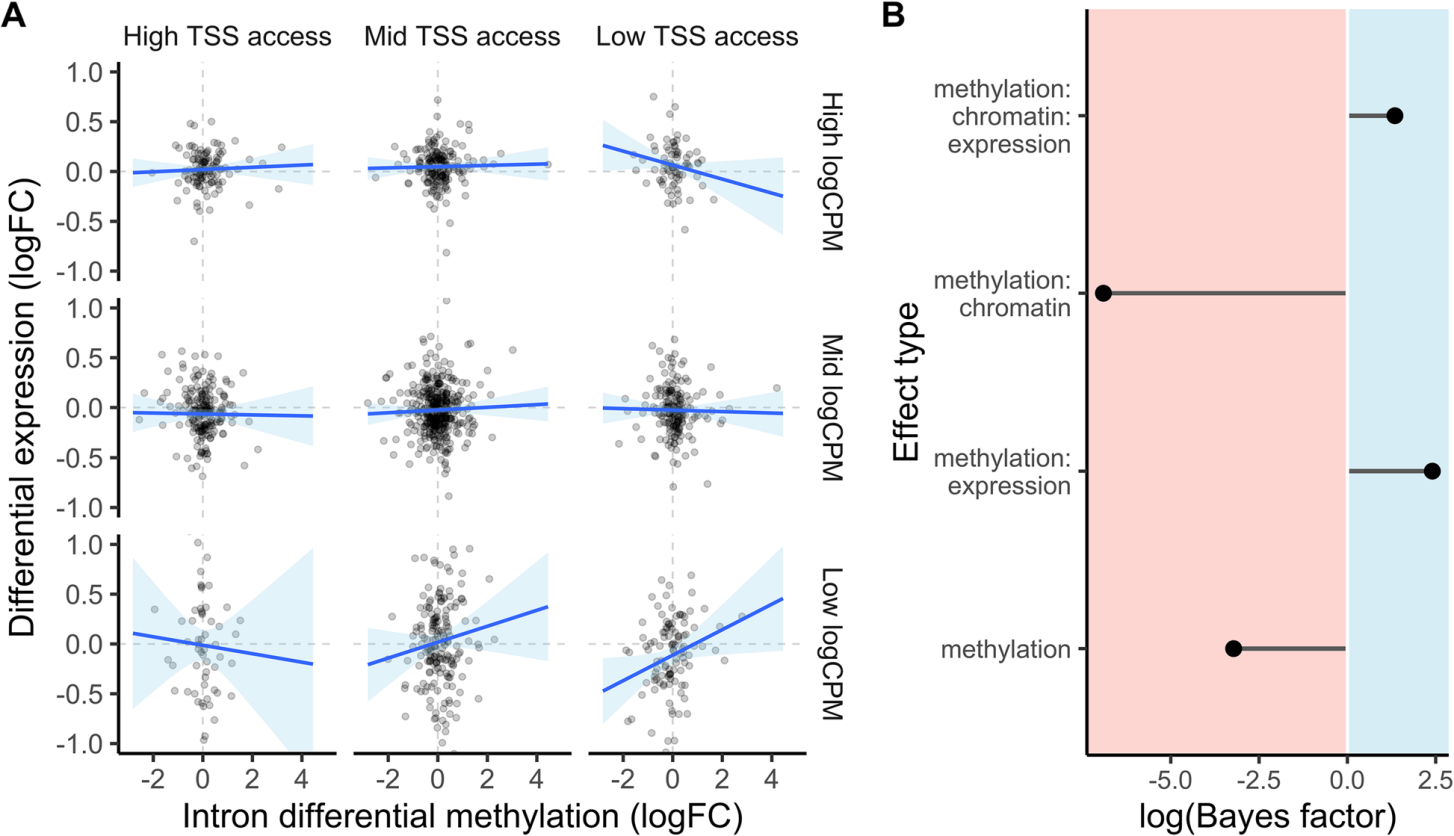
Differential intron methylation affected expression conditional upon TSS accessibility and transcript abundance. **A** Differential gene expression in response to maternal upwelling is plotted against mean intron differential methylation. Rows and columns group data based on transcript abundance and transcriptional start site (TSS) accessibility quartiles. First and last rows/columns denote highest and lowest quartiles while the middle row/column denote second and third quartiles. **B** Log-scale inclusion Bayes factors depicting the probability of observed data under models with a parameter relative to those without it, including the interaction effect visualized in **A** (“methylation:chromatin:expression”). Positive Bayes factor values represent parameters that improved predictive strength across models and were jointly included in the selected model of differential expression responding to maternal conditioning. For parameters listed on the *y*-axis, “methylation” = intron differential methylation, “chromatin” = TSS accessibility, and “expression” = baseline gene expression (logCPM). *n* = 12 RNA-seq and RRBS replicate libraries; *n* = 3 ATAC-seq replicate libraries

**Fig. 4:**
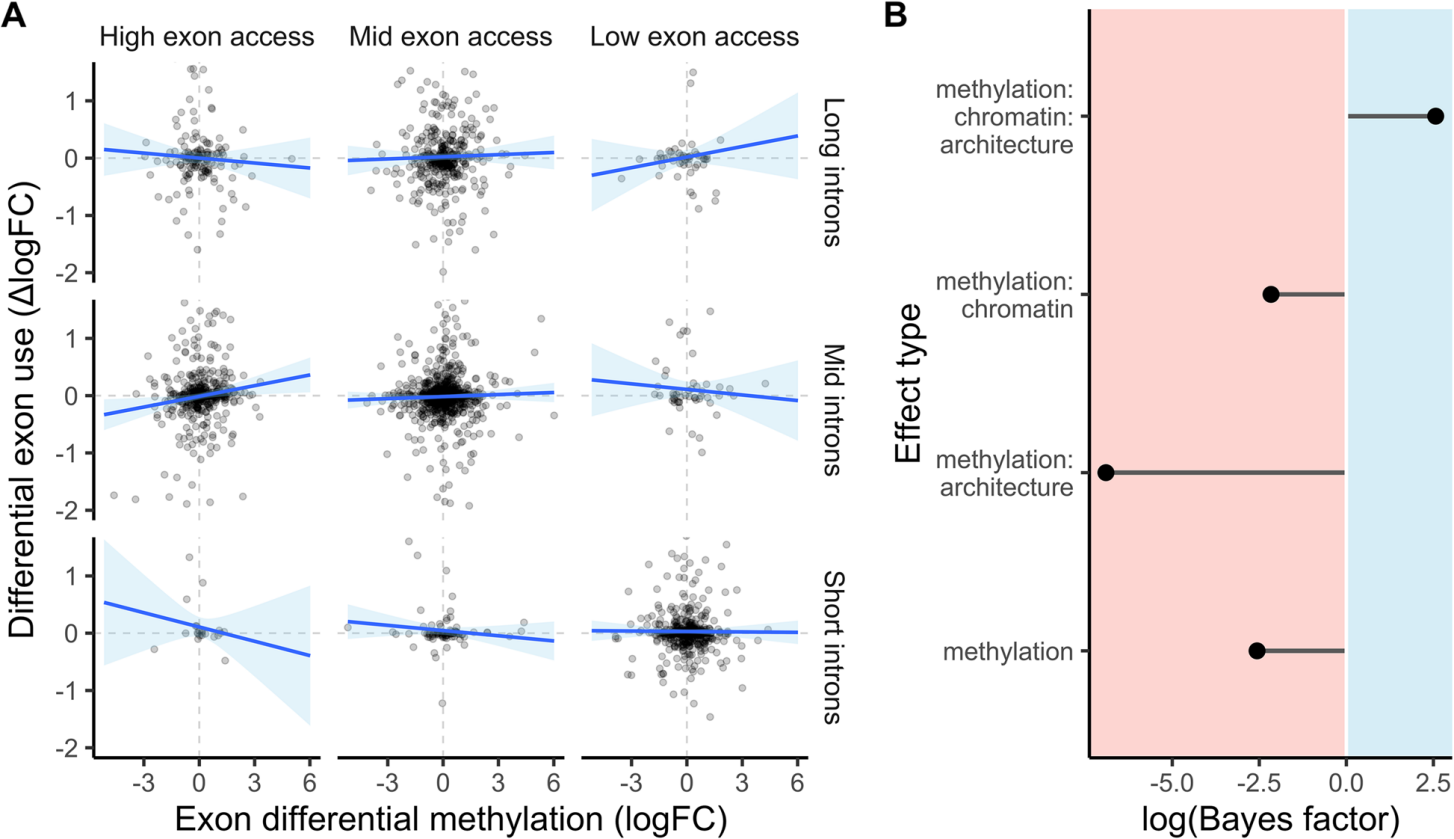
Differential exon methylation affected splicing conditional upon exon accessibility and genic architecture. **A** Differential exon use in response to maternal upwelling is plotted against exon differential methylation. Rows and columns group data based on total genic intron length and exon accessibility quartiles. First and last rows/columns denote highest and lowest quartiles while the middle row/column denote second and third quartiles. **B** Log-scale inclusion Bayes factors depicting the probability of observed data under models fitting a parameter relative to those without it, including the interaction effect visualized in **A**. For parameters listed on the *y*-axis, “methylation” = exon differential methylation, “chromatin” = exon accessibility, and “architecture” = total genic intron length. *n* = 12 RNA-seq and RRBS replicate libraries; *n* = 3 ATAC-seq replicate libraries

Our results demonstrated (i) that differential DNA methylation was associated with gene regulation during TGP in *Strongylocentrotus purpuratus* and (ii) that these effects were conditional upon chromatin accessibility and genic architecture. We observed positive correlations between baseline DNA methylation, transcript abundance, and the occurrence of alternative splicing (i.e., the average level of each value for a given gene, controlling for environmental variation) as shown in Fig. 1. With regard to plastic changes in DNA methylation and gene regulation induced by maternal environments (Fig. 2), associations between differential gene body methylation and differential gene expression or splicing were affected by interactions between differential methylation, chromatin accessibility, and genic architecture: the strength and direction of DM’s effects were contingent upon these additional genomic and accessibility states (Figs. 3 and 4). We describe these results in three sections below, focusing first on baseline relationships between DNA methylation and transcription followed by epigenetic and gene regulatory responses to experimental upwelling. Finally, we present the results of integrated epigenomic models of DNA methylation’s association with gene regulation during TGP. We also quantified the intra- and interexperimental variation in ATAC-seq measures of chromatin accessibility in early-stage *S. purpuratus* (Additional file 1: Fig. S1) in order to ensure robust integration of functional genomic data between studies.

### Associations between constitutive epigenomic states and transcription

GBM in *S. purpuratus* prism larvae measured via RRBS showed significant and positive correlations with gene expression level and the probability of alternative transcriptional variants. Mean promoter methylation demonstrated a significant, albeit weak, negative effect on expression (logCPM). Mean exon and intron methylation both exhibited stronger, positive effects on expression (Fig. 1A). Genes with high levels of intron or exon methylation were more likely to exhibit transcript variants consistent with alternatively spliced isoforms, alternative TSS, and/or exon skipping. Here, the presence of alternative transcript variants serves as a measure of baseline alternative splicing: a binary variable for whether a gene possesses annotations for multiple isoforms. This baseline measure of splicing is distinct from alternative splicing in response to environmental variation. The relationship between the probability of transcript variants and promoter methylation was insignificant (Fig. 1B). Significance was determined using a probability of direction test, a Bayesian corollary of the *p*-value, and is further described under the “Methods” section [59].

Chromatin accessibility at TSS, exons, and introns was correlated with gene expression, but not with the probability of transcriptional variants. Chromatin accessibility was enriched proximal to TSS (Fig. 1C) but was greatest in introns, which exhibited a mean of 0.19 ± 0.11 ATAC-seq reads per bp compared to 0.043 ± 0.001 and 0.039 ± 0.043 in TSS and exons, respectively. Open chromatin regions showed ~ 30% less CpG methylation than inaccessible regions (Fig. 1D). Chromatin accessibility within ± 500 bp of TSS and gene bodies was significantly and positively correlated with gene expression, with TSS accessibility exhibiting an effect that was 32.98% stronger than gene body accessibility (Additional file 1: Fig. S2). TSS accessibility was also positively correlated with the length of first exons (e.g., distance of first intron to TSS) as shown in Additional file 1: Fig. S3 and uncorrelated with GBM (Additional file 1: Fig. S4). CpGs within − 1 kb promoters, exons, and introns exhibited mean methylation levels of 34.70%, 43.21%, and 44.64%, respectively. Although a strong decline in DNA methylation was observable at CpGs proximal to accessible chromatin regions, average gene-level intron and exon methylation showed no relationship with chromatin accessibility of either introns, exons, or TSS (Additional file 1: Figs. S2 and S4).

Regarding the effect of GBM on baseline gene expression, exon and intron methylation also exhibited a significant, antagonistic interaction such that genes with high methylation at both introns and exons were not more expressed than genes with high methylation at only introns or exons. Interestingly, accounting for TSS accessibility in models of logCPM resulted in the loss of a significant effect of intron methylation on gene expression. In addition to chromatin accessibility’s correlation with gene expression, TSS accessibility also exhibited a significant and positive correlation with the probability of alternative transcriptional variants. This effect was insignificant however after accounting for GBM. Thus, GBM at exon and introns was the only significant predictor of alternative splicing events.

### Transcriptional and epigenetic responses to environmental variation

Maternal and developmental exposure to experimental upwelling induced changes to gene expression level (differential expression or “DE”) as well as differential exon use (DEU), a measure of alternative splicing and exon skipping calculated by subtracting the logFC of DE in one exon from its corresponding transcript (Fig. 2). Developmental upwelling exposure induced 2263 upregulated and 2459 downregulated, differentially expressed genes (DEGs). Maternal exposure induced 1380 upregulated and 1025 downregulated DEGs (Fig. 2). After applying a log_2_FC cutoff of > 1.0, 309 significant developmental DEGs were retained while 245 maternal DEGs were retained. Absolute logFCs of DE among maternal DEGs were significantly higher than developmental DEGs by 10.45%.

Significant DEU was evaluated using both gene- and exon-level tests. Developmental upwelling induced 78 alternatively spliced genes (ASGs) while maternal upwelling induced 121 ASGs, with 16 ASG genes shared between treatments (Additional file 1: Fig. S5). Significant DEU was detected among 44 exons in response to developmental upwelling: 14 upregulated or “included” exons and 30 downregulated or “dropped” exons. DEU induced by maternal upwelling occurred in 47 exons: 12 included and 35 dropped exons (Fig. 2). Maternal and developmental DE and DEU predictions are available in Additional file 2. Gene ontologies enriched among DE and DEU genes can be found in Additional file 3.

In response to maternal upwelling, 288 CpGs were hypermethylated and 233 were hypomethylated. 0 CpGs were differentially methylated in response to developmental upwelling unlike DE and DEU, which both exhibited greater or equal variation in response to developmental versus maternal exposure (Fig. 2). Among genic features, maternal upwelling induced hypermethylation at 6 promoters, 75 introns, and 32 exons and hypomethylation at 6 promoters, 57 introns, and 25 exons. Developmental upwelling exposure induced hypermethylation at 4 promoters, 19 introns, and 2 exons and hypomethylation at 0 promoters, 4 introns, and 0 exons. Because of the limited effect of developmental conditioning on DM, we exclusively focus on maternal effects for the remainder of the results section.

Forty-three and 49 genes were both differentially expressed and differentially spliced in response to developmental upwelling and maternal upwelling, respectively (Additional file 1: Fig. S5). The molecular function (MF) GO terms ‘structural molecule activity’ and ‘structural constituent of ribosome’ and the biological process terms ‘obsolete GTP catabolic process,’ ‘small GTPase mediated signal transduction,’ and ‘cellular amide metabolic process’ were enriched among differentially spliced exons in response to both maternal and developmental treatments. Exons differentially spliced under the maternal treatment were also enriched for the biological processes (BP) ‘cellular localization’ and ‘nuclear transport’ among others. All enriched gene ontologies among DE and DEU genes are described in Additional file 3. Additionally, functional enrichment among differentially methylated genes is extensively described in Strader et al. 2020 [15].

### Correlations between differential methylation and transcriptional responses to environmental variation were shaped by chromatin state and genic architecture

A Bayesian model fitting and selection workflow was employed to robustly test for effects of DM, chromatin accessibility, and genic architecture on DE and DEU induced by maternal upwelling exposure during gametogenesis. Described in greater detail under Materials and Methods, this workflow reduced overfitting, false positive predictions, and aimed to promote reproducibility of the results below. This was achieved by fitting multiple, iterative models of DE and DEU before performing model selection, parameter selection, and intensive quality checks of model predictions. For accurate interpretation of the results, it is important to state that models of DE and DEU incorporating ATAC-seq measures of chromatin accessibility were limited to testing for an effect of baseline accessibility on the outcome variable rather than differential accessibility. This is because ATAC-seq libraries were sourced from a publicly available dataset. Here, chromatin accessibility should be interpreted as a baseline measure as defined above: an expected level of chromatin accessibility, controlling for the effects of environmental variation.

The strength of associations between differential GBM and DE or DEU induced by maternal environment were dependent on chromatin accessibility within genic regions. Differential GBM across whole genes did not affect DE, while intron DM was significantly associated with DE. The selected model of DE as a function of intron DM under maternal upwelling included a significant three-way interaction between intron DM, TSS accessibility, and logCPM. Intron DM had higher absolute effects on DE among genes with poorly accessible TSS. These effects were positive among genes with low expression and negative for those that were highly expressed, treating both expression level (logCPM) and TSS accessibility as continuous effects. For the sake of visualization, Fig. 3A shows this result using bins of logCPM and TSS accessibility rather than representing each variable as continuous. An inclusion Bayes factor test demonstrated that observed DE was 3.84 × more likely under models that fit this interaction between intron DM, logCPM, and TSS accessibility relative to models lacking this parameter (Fig. 3B). Figure 3B visualizes the relative likelihood of observed DE across models with and without a given parameter including this three-way interaction and a two-way interaction between intron DM and logCPM, both of which were included in the selected model. The selected model also predicted a significant, negative, effect of constitutive intron methylation on DE (Additional file 1: Table S1). Model and parameter selection selected against singular, direct effects of intron DM (Fig. 3B). Models fit with predictors for DM at promoters, first introns, and exons yielded null effects of methylation at these features on DE.

Some model iterations in the top 25% of marginal likelihoods indicated that intron length interacted with intron DM to affect expression. Intron hypermethylation of genes in the top quartile of intron length silenced expression while genes in the middle-to-lowest quartiles of intron length showed enhanced expression as a result of intron hypermethylation (Additional file 1: Fig. S6), though the inclusion Bayes factor for this interaction was negligible (BF = 1.67). Information regarding the selected model of DE induced by maternal conditioning is available in Additional file 1: Table S1 and Additional file 1: Figs. S7–S12.

A Fisher's exact test demonstrated that genes in the lowest logCPM quartile and lowest quartile of TSS accessibility were enriched with MF GO terms that included ‘nucleotidyltransferase activity’ and ‘cytoskeletal motor activity,’ two MF terms that were also enriched among genes with CpGs that were differentially methylated under maternal upwelling [15]. The BP GO terms ‘plasma membrane bounded cell projection assembly’ and ‘movement of cell or subcellular component’ were also enriched among genes with low TSS accessibility and expression, among others (Additional file 3). Genes in the lowest TSS accessibility quartile and highest logCPM quartile were enriched with the MF terms ‘threonine-type endopeptidase activity,’ ‘transcription regulator activity,’ and ‘calcium ion binding’.

Differential methylation at singular exons induced by maternal upwelling interacted with gene body accessibility and genic architecture to affect DEU. Selected models of DEU yielded a significant three-way interaction between exon DM, exon accessibility, and total genic intron length. Positive correlations between exon DM and DEU were observed among exons from genes with poor exon accessibility while negative correlations were observed among exons from genes with high exon accessibility. The absolute effect strength of exon DM on DEU was stronger among genes with longer introns (Fig. 4A). The inclusion Bayes factor of the interaction between exon DM, exon accessibility, and intron length equaled 13.03 (Fig. 4B). The selected model of DEU also predicted a significant, positive effect of exon number on DEU and a singular, negative effect of exon length (Additional file 1: Table S2). Additional predictions and quality metrics for selected models of DE and DEU are available in Additional file 1: Figs. S7–S18.

Genes in the lowest quartile of exon accessibility and highest intron length quartile were enriched with the MF GO terms “calcium ion binding,” “cytoskeletal motor activity,” and “ATPase activity,” among others, and the BP terms “purine-containing compound metabolic process”, “microtubule-based movement,” and “cell adhesion.” Genes with long introns and high exon accessibility were also enriched with the MF term “calcium ion binding,” as well as “transporter activity,” “small molecule binding”, and others and enriched BP terms including “cell adhesion,” “localization”, “regulation of intracellular signal transduction”, and others (Additional file 3). Information regarding the selected model of DEU induced by maternal conditioning is available in Additional file 1: Table S2 and Additional file 1: Figs. S13–S18.

## Discussion

We sought to characterize relationships between differential methylation and transcriptional plasticity in the purple urchin *Strongylocentrotus purpuratus*, a species for which DNA methylation appears to play a role in transgenerational plasticity [15, 48, 50]. Differential gene body methylation in *S. purpuratus* larvae induced by maternal upwelling exposure (low temperature and high *p*CO_2_) exhibited significant and strong effects on differential expression and differential exon use among subsets of genes contingent upon chromatin accessibility and genic architecture. These results support the hypotheses that differential methylation induced during TGP elicits multiple gene regulatory effects in *S. purpuratus* and, secondly, that these effects are conditional upon the chromatin state and genic feature at which DM occurs. Here we interpret our results in the context of *S. purpuratus* and invertebrate physiological ecology before highlighting questions and approaches to be pursued in future studies of ecological epigenomics in metazoans. Because developmental conditioning to upwelling induced limited differential methylation, we restrict our discussion of environmental changes in DNA methylation and its relationship with differential gene expression to the effects of maternal conditioning.

### Relationships between DNA methylation, chromatin accessibility, and gene expression in *Strongylocentrotus purpuratus* and other invertebrates

Baseline patterns of genomic methylation, chromatin accessibility, transcription, and the relationships between these processes in *S. purpuratus* were largely consistent with those typically observed in other invertebrates, particularly our observation that GBM correlated with transcript abundance [19, 26, 27, 31–33, 60–65]. GBM inhibition in metazoans can reduce gene expression, with some evidence of causal links between GBM and expression [36, 66, 67]. One potential link between invertebrate GBM and gene expression could be that when intragenic DNA methylation is bound by methyl-DNA-binding domain protein 2/3, acetyltransferases are recruited to promote H3K27 acetylation and transcriptional elongation as evidenced by empirical study in insects [68]. A non-competing hypothesis suggests that GBM correlates with gene expression to support sequence conservation and transcriptional homeostasis. For example, genes with intragenic hypermethylation are less accessible in at least some invertebrates [31], which can protect them from mutation [69] and slow evolutionary rates across invertebrate lineages [70–73].

Intron methylation was also positively correlated with the prevalence of transcript variants among *S. purpuratus* genes. This is consistent with associations between GBM and alternative splicing in other metazoans. Intragenic hypermethylation recruits methyl CpG binding protein 2 (MeCP2) to splice junctions, which promotes exon recognition and, in some cases, intron retention via alterations to elongation rate [74, 75]. Our results shed additional light on patterns observed in honeybees by Flores et al. that methylated genes exhibit more alternative splicing events and those by Libbrecht et al. in ants demonstrating that included introns have higher levels of methylation than expected [60, 62]. Further investigation into the strength of intron methylation’s relationship with transcript variants in *S. purpuratus* relative to other genic features may better reveal the mechanisms underpinning GBM’s association with alternative splicing.

Models of logCPM that included TSS or exon accessibility as predictors demonstrated significant, positive correlations with gene expression. However, variables related to chromatin accessibility were not selected for inclusion in models of logCPM, indicating their low predictive power. Chromatin accessibility was not associated with the occurrence of transcriptional variants and splice isoforms, but it is difficult to compare this result with other case studies because these two processes have not been frequently studied together in invertebrates. Compared to its association with constitutive levels of expression and splicing, ATAC-seq estimates of chromatin accessibility provided greater predictive power in models of DE and DEU induced by environmental variation.

### Differential methylation’s associations with gene regulation depend on chromatin state and genic architecture

Our finding that DM induced by maternal environment was associated with DE and DEU conditional upon chromatin accessibility and genic architecture supports the hypotheses that (i) DM is associated with gene regulation in invertebrates during plastic responses to the environment and (ii) that these correlations are contingent upon chromatin accessibility and genic architecture. DE genes exhibited little overlap with DEU (Additional file 1: Fig. S5), but both molecular responses to environmental stress were predicted to correlate with interacting epigenomic processes. This degree of complexity underlying differential GBM’s functions juxtaposes the simple relationship between promoter DM and expression exhibited across vertebrates [76]. Such complexity is expected, however [77], and our evidence helps to resolve null one-to-one correlations between differential GBM and gene expression observed in prior studies: differential GBM bears multiple associations with gene regulatory processes in *S. purpuratus*, but these associations only exist among subsets of genes based on chromatin accessibility and the architecture of gene bodies. To our knowledge, our study is the first to detect that associations between differential expression and methylation induced by abiotic environments depend on chromatin accessibility. While a great deal remains to be uncovered about the gene regulatory roles of GBM, our results aid in understanding several key areas regarding epigenomic regulation of phenotypic plasticity and molecular responses to environmental stress.

Models of invertebrate environmental adaptation or acclimatization may not be improved by accounting for singular, independently acting chromatin modifications. Studies that aim to link DNA methylation to gene expression or phenotype across experimental treatments, natural populations, or generations via inheritance may suffer from poor predictive ability without accounting for the genomic features and chromatin states at which DM is occurring. While bisulfite sequencing has rapidly increased in application to experimental and natural studies of environmental adaptation [78, 79], methods that permit for the simultaneous quantification of chromatin accessibility and DNA methylation such as ATAC-Me [80] may offer greater promise for identifying functional epigenomic patterns when combined with RNA-seq. The need for integrated epigenomic studies in metazoans, and ecology and evolution at large, should not come as a surprise. Even in plants and mammals for which DNA methylation’s regulatory roles are better understood, its associations with gene expression can be tenuous at whole-genome levels [1, 81] and can depend on chromatin state [14]. For example, correlations between CpG DM and DE between mammalian cell types are stronger among genes that also exhibit differential chromatin accessibility between cells [40]. The exact process underpinning interactions between DNA methylation and chromatin accessibility shaping expression in *S. purpuratus* during TGP remain unclear, but several results related to mechanism are worth highlighting.

Associations between differential GBM and DE were strongest when intron DM occurred in genes with low TSS accessibility (Fig. 3A). Introns play a significant role in enhancing gene expression and can be essential to transcriptional initiation at canonical 5′ TSS [82, 83] or spur alternative, downstream initiation sites when first exons are distally positioned [83]. In vertebrates, first intron methylation is often negatively correlated with gene expression [34]. Introns’ contributions to gene regulation can also depend on chromatin modifications in vertebrates: length of the first exon (e.g., distance of first intron from TSS) is negatively correlated with activating chromatin marks at promoters such as H3K4me3 and H3K9ac [84]. In *S. purpuratus*, however, we found that (i) methylation of first introns did not silence expression and (ii) distance of the first intron from TSS was positively correlated with TSS accessibility (Additional file 1: Fig. S3), suggesting that different processes linking intron methylation to gene expression are likely at play compared to known mechanisms in vertebrates.

Introns possess both enhancing and silencing effects on gene expression across eukaryota [85]. Among genes with inaccessible TSS that exhibited correlations between intron DM and DE, the direction of this effect also depended on gene expression level: DE of highly expressed genes negatively correlated with DM while this correlation was positive for genes with low expression (Fig. 3A). Experimental demethylation of gene bodies [35] and whole genomes [36] has resulted in a mixture of significant up- and down-regulation in eukaryotes. It is plausible that differential GBM will induce upregulation or downregulation depending on the intragenic regulatory elements existing within gene bodies and how they interact with DNA methylation. The representation of enhancing and silencing regulatory elements among genes possessing positive or negative correlations between DM an DE should be further investigated.

Differential GBM in *S. purpuratus* larvae interacted with chromatin state and genic architecture to potentially influence alternative splicing and/or exon skipping. A three-way interaction between exon DM, exon accessibility, and genic intron length affected DEU such that (i) the absolute effect of exon DM was strongest among genes with greater total intron lengths and (ii) exon accessibility altered the direction of this effect. To our knowledge, our results mark the first evidence in an invertebrate of a significant association between DM and exon inclusion responding to environmental variation. Associations between DM and DEU underscore the potential for DNA methylation’s impact on environmental responses to be the additive result of associations with different modes of gene regulation.

The positive correlation between exon DM and DEU in genes with inaccessible exons was expected as hypermethylation at alternatively spliced exons has generally been associated with their inclusion in transcripts [60, 86]. The effect of total intron length on DEU is also consistent with alternative splicing’s pervasiveness among longer genes [60, 87], whose size we found is largely attributable to intron length in *S. purpuratus*. Chromatin accessibility at exons may influence the direction of DM’s association with DEU and splicing because chromatin state and DNA methylation are both known to influence exon inclusion and skipping [74, 86, 88]. Positive or negative associations between DM and exon use can manifest depending on the intragenic regulatory elements present within a gene, which are often associated with either accessible or inaccessible chromatin states, and their corresponding cofactors that may induce exon inclusion or skipping [89, 90].

Mixtures of positive and negative associations between DM and DE or DEU may be obscured by genome-wide regressions of DE and DM performed in prior studies. Such tests assume that a unidirectional effect of DM on DE should be evident across the whole transcriptome. Looking forward, methods permitting gene-wise rather than genome-wide tests of environmentally induced DM’s effect on DE such as mediation analysis [91] and network-guided multivariate regressions [92, 93] may provide a more resolved understanding of DNA methylation’s association with transcriptional responses to the environment. Mediation analysis has been performed using epigenetic and phenotypic data [94, 95] and can expand on traditional DE tests to estimate both the direct effect of an environmental variable on gene expression and indirect effect of environment on expression mediated by changes in DNA methylation. These methods can also incorporate indirect effects of chromatin accessibility measured across experimental replicates.

Correlated changes in DNA methylation and gene regulation could also be explained by mechanisms alternative to a causal effect of DNA methylation on expression. For example, evidence of DE preceding DM exists across plants and animals in a variety of contexts. Following bacterial infection in human dendritic cells, the majority of changes in DNA methylation occurred after DE of corresponding genes [96]. In rice, DM proximal to genes exhibiting DE under nutritional stress largely followed changes in gene expression [97]. If we assume the hypothesis that DE in *S. purpuratus* induced by maternal conditioning triggers changes in GBM, our evidence of interactions between chromatin state and DM should be interpreted differently. As discussed by Pacis et al. and others, DM that follows DE can potentially prime promoters or enhancers to block or be bound by *trans*-acting regulatory elements during secondary responses to environmental change that follow initial DE. DM may also serve to return DEGs to normal levels following acute responses or stabilize the expression of differentially regulated genes, consistent with GBM’s role in transcriptional homeostasis [19, 31, 98]. Ecological epigenomic research in *S. purpuratus* and other metazoans may better resolve the temporal coupling of DNA methylation and gene expression by integrating bisulfite sequencing and RNA-seq with time series experiments. Assuming this study were replicated with incorporation of ATAC-seq in maternal upwelling and non-upwelling treatments, it is plausible if not likely that our reported results would vary due to the inclusion of differential accessibility as a fixed effect in addition to baseline accessibility, which we achieved here. It could be the case that variance explained by baseline accessibility is better predicted by differential accessibility as more accessible regions of the genome are more likely to vary in that accessibility [99]. Alternatively, both baseline and differential accessibility could have distinct singular effects or interactive effects associated with gene regulatory responses to environmental change.

## Conclusions

Variation in DNA methylation appears to be a component of molecular responses by many metazoans to predicted global change including ecologically critical, threatened groups such as stony corals [21] and polar pteropods [100] or detrimental invasive species [101]. Given the potential heritability of DNA methylation in some clades [102, 103], exacting its transcriptional and phenotypic consequences is critical for understanding the mechanistic basis of TGP. Our findings (i) provide quantitative support for the hypothesis that gene regulation by differential gene body methylation in *S. purpuratus* is affected by chromatin accessibility and genic architecture and (ii) indicate that these effects influence both gene expression and mRNA splicing. The majority of ecological epigenomic studies in metazoans have focused on the singular effects of differential methylation on expression. However, DNA methylation is not a silver bullet to predict transcriptional changes by *S. purpuratus* in response to environmental variation. Rather, it is likely one cog in the epigenomic machinery contributing to plasticity in gene expression and alternative splicing. A shift toward integrated studies combining DNA methylation, chromatin accessibility, and genomic/genic architecture may be necessary to accurately quantify non-genetic sources of transcriptional and phenotypic variation in invertebrates and other eukaryotes.

## Methods

The methods applied in this study served to achieve three aims: (i) evaluating relationships between baseline DNA methylation (i.e., constitutive methylation level), chromatin accessibility, and gene expression by integrating RNA-seq, bisulfite-seq, and ATAC-seq data from prism-stage *S. purpuratus*, (ii) testing for effects of maternal and developmental conditioning on differential methylation, expression, and splicing, and (iii) modeling differential expression and splicing induced by maternal conditioning as a function of variation in methylation and chromatin accessibility.

### Data sources

For RNA-seq and bisulfite sequencing datasets, a controlled transgenerational experiment was performed [15]. Briefly, adult urchins were conditioned to two treatments, non-upwelling (631 ± 106 μatm *p*CO_2_ and 16.8 ± 0.2 °C) and upwelling (1390 ± 307 μatm *p*CO_2_ and 12.7 ± 0.5 °C), mimicking variation in their natural environment [46]. Temperature and *p*CO_2_ conditions were maintained by a flow-through CO_2_ system [104] and described in detail by Strader et al. 2020 [15]. Treated seawater was evenly pumped from two reservoir tanks to conditioning tanks at a rate of 20 L/h. Adult urchins were induced to spawn and fertilizations were performed in ambient seawater conditions using 1 non-upwelling male and pooled eggs from 9 females per treatment. Embryos were reared in the same conditions as their parents or the reciprocal condition in triplicate using a flowthrough system with seawater treated as described above. 

Once larval development progressed to the early prism stage, pooled samples from 12 different culturing replicates containing 6000 larvae per pool were collected for RNA-seq and RRBS and flash frozen in liquid nitrogen before storage at − 80 °C. RRBS is less biased across genomic features relative to other reduced representation bisulfite sequencing methods [105]. Libraries for polyA-enriched RNA-seq and RRBS were constructed at the UC Davis genome center and sequenced on the Illumina HiSeq 4000 (BioProject: PRJNA548926). RRBS and RNA-seq libraries were separately sequenced on two lanes of Illumina HiSeq 4000 using single end 100 bp reads (*n* = 12 samples per lane). RNA-seq libraries averaged a size of 29.72 million raw reads ± 1.60 million SD and a mean mapping efficiency of 62.11% ± 1.89% SD. RRBS libraries averaged a size of 24.79 million raw reads ± 4.65 million SD and a mean mapping efficiency of 38.13% ± 0.62% SD. For more information on RNA-seq and RRBS libraries' quality and specifications, please see Additional file 4.

ATAC-seq data was obtained through the Gene Expression Omnibus (BioProject: PRJNA377768). For integration with the Strader et al. datasets, we chose ATAC-seq profiles for animals at 39 h post-fertilization, the closest developmental time point for early prism larvae, for which 3 pooled samples were sequenced (GSM2520650, GSM2520651, GSM2520652). ATAC-seq bed files were concatenated and summarized using the R package *ChIPSeeker* v1.22.1 [106] to quantify chromatin accessibility as the mean density of Tn5 ATAC-seq reads per replicate per bp. In order to evaluate the applicability of the PRJNA377768 ATAC-seq dataset to our own experiment, we measured interexperimental variation in ATAC-seq peaks identified in early-stage *S. purpuratus* [107, 108]. We found that counts of ATAC-seq peaks detected between different experiments sequencing the same life history stages were strongly correlated as shown in Additional file 1: Fig. S1 (slope of 0.89; *R*^2^ = 0.80; *p* < 0.0001) and comparable to within-experiment variation between replicates (slope = 0.80–0.93; *R*^2^ = 0.65–0.87; *p* < 0.0001). These results suggest that integrating ATAC-seq reads from PRJNA377768 with RRBS and RNA-seq data from our own experiment is robust to interexperimental variation.

Mean chromatin accessibility of ± 500 bp transcriptional start sites (TSS), introns, and exons were each calculated in both gene- and feature-wise manners for downstream analyses by counting ATAC-seq reads from all three .bed replicates per genomic feature, dividing the total reads-per-feature by feature length to estimate ATAC-seq read density, and dividing the total density by 3 in order to calculate mean density.

The sequencing approaches used for data sourced in this study were suited to downstream analyses of gene regulation such as differential exon use and genomic feature-specific changes in DNA methylation. The use of polyA-enriched RNA-seq libraries is beneficial for analyzing alternative splicing as it mitigates the contribution of unprocessed RNA to quantification of differential exon use [109]. RRBS poses fewer biases on CpG representation across genomic feature type relative to other reduced representation BS-seq methods [105].

### Evaluation of ATAC-seq integration

Although ATAC-seq datasets are commonly integrated from one experiment into another, interexperimental variation in chromatin accessibility measured via ATAC-seq was assessed among early life stage *S. purpuratus* in order to assess the suitability of integrating publicly available ATAC-seq data within this study. Multiple prism-stage ATAC-seq datasets do not exist, prohibiting such a test on the stage examined in this study. Rather, chromatin accessibility peaks have been identified in 20 h post-fertilization (hpf) and 24 hpf *S. purpuratus* blastulas: GSE160461 and GSE96927 [107, 108, 110, 111]. Because of differences in sampling time point, sequencing parameters, and bioinformatic pipelines, these two datasets are likely to serve as an underestimation of variance in chromatin accessibility between experiments.

.bed files of called peaks for 2 biological replicates from 20 hpf blastulas (GSE160461) and 4 .bed files from 24 hpf blastulas representing isolated primary mesenchyme cells or all other cells (GSE96927) were input into R and converted to Genomic Ranges objects using GenomicRanges v1.38.0 [112]. The genomic locations of peaks from both GSE160461 20 hpf replicates and the 2 GSE96927 “other” 24 hpf blastula cell type replicates were annotated using ChIPSeeker v1.22.1 [106] before counting the number of peaks present within ± 500 bp TSS, exons, and introns of all CDS within the *S. purpuratus* v3.1 genome build. Using this dataset of genome-wide TSS, exon, and intron ATAC-seq peak counts from both experiments, a linear regression was fit to mean-standardized peak counts per feature in GSE160461 as a function of standardized peak counts in GSE96927. Accessibility measured as the standardized number of identified ATAC-seq peaks were strongly correlated between the two experiments (Additional file 1: Fig. S1). With a slope of 1.00 representing a 1:1 correlation, this regression fit a slope of 0.89; *R*^2^ = 0.80; *p* < 0.0001. This between-experiment correlation is comparable to intraexperimental variation between replicates from GSE96927 (slope = 0.93; *R*^2^ = 0.87; *p* < 0.0001) and is in fact stronger than intraexperimental correlation in GSE160461 (slope = 0.80; *R*^2^ = 0.65; *p* < 0.0001).

Pairwise overlaps between genomic ranges of replicates from each experiment were then identified using the findoverlappairs() function from GenomicRanges [112] using two overlap distances: − 1 bp and + 500 bp. The number of overlaps was then calculated as the proportion of peaks in the smaller dataset (GSE96927) with at least one overlap in the opposite dataset. The number of overlaps was calculated across all reciprocal comparisons between replicates from the two experiments: 2 reps × 4 reps for 8 comparisons total. The mean number of overlaps across all 8 contrasts was found to be 72.16 ± 6.15% when overlap distance was set to + 500 bp and 50.03 ± 7.33% with an overlap distance of − 1 bp.

These results demonstrate that ATAC-seq measures of chromatin accessibility are reproducible at the scale of genomic features, with some but substantially less reproducibility at the scale of singular peaks. In fact, between-experimental correlations in feature-wide accessibility were comparable to within-experiment correlations between replicates. Therefore, the approach taken in this study to fit models of gene expression with parameters representing genewise levels of accessibility at TSS, exons, and introns taken from ATAC-seq data generated in a separate experiment is robust to potential interexperimental variation.

### Gene expression analyses

RNA-seq reads were trimmed of adaptor sequences and filtered for quality using *TrimGalore*. Cleaned reads were mapped to the “Spur_3.1” genome assembly using *hisat2* [113]. Gene and exon counts were compiled with *featureCounts *[114]. Gene- and exon-level read counts were filtered to retain genes with > 0.5 counts per million (CPM) across ≥ 75% of samples and analyzed in *edgeR* v3.28.1 [61] in order to estimate differential expression and differential exon use, a measure of exon inclusion or exclusion attributable to skipping and splicing. DEU was selected as a measure of alternative splicing as opposed to differential isoform expression because this study leveraged 100 bp single end RNA-seq libraries rather than long read RNA-seq, which is necessary for performing isoform-level analyses of gene expression data [115, 116]. Furthermore, downstream analyses integrating DNA methylation and measures of alternative splicing were conducted at the exon rather than isoform level, and thus, DEU’s robust measure of splicing and exon skipping [117] was appropriate for this study’s scope and questions.

To test for differential expression and differential exon use, gene- and exon-level counts were modeled as a function of maternal environment, developmental environment and their interaction using the robust iteration of the *edgeR* glmQLfit function to fit negative binomial generalized linear models (GLMs). DEU was assessed by applying the edgeR function diffSpliceDGE to exon-level negative binomial GLMs, which outputs exon use coefficients denoted as ΔlogFC: exon logFC–gene logFC [118, 119]. To ensure that these tests were specific to alternative splicing and exon skipping, exon-level read count data were filtered to remove transcripts that exhibited DEU consistent with changes in spurious transcription marked by gradual reductions in exon use toward 5′ regions of a transcript. Significant DE and DEU was determined using FDR-adjusted *p*-values (alpha = 0.05). Enriched gene ontologies (GO) were identified among genes exhibiting DE or DEU with Mann–Whitney *U* tests input with signed, -log *p*-values using rank-based gene ontology analysis [120].

Robust negative binomial dispersion estimates were calculated using empirical Bayesian shrinkage with the *edgeR* function estimateGLMRobustDisp. Log_2_ foldchanges (logFC), *F*-statistic scores, and *p*-values for genewise DE between maternal and developmental treatments were estimated using the *edgeR* function glmQLFTest to account for uncertainty of tagwise dispersion estimates and improve type I error control.

Quantifying DEU attributable to alternative splicing and exon skipping required the removal of genes exhibiting patterns of exon use consistent with spurious intragenic transcription and alternative TSS. Genes that are spuriously transcribed or exhibit alternative TSS possess exons with progressively lower inclusion toward 5′ ends [19]. Filtering out such genes from exon-level read counts used in DEU analysis required fitting linear models to exon-use data as a function of exon number and removing genes with positive slopes and a *y*-intercept of DEU >  − 0.25. Without this filtering step, 56.0% of genes that exhibited significant DEU under maternal upwelling would likely have been attributed to alternative TSS or spurious transcription while such genes would have composed 64.9% of significant DEU under developmental upwelling. While this approach targeted DEU attributed to splicing and exon skipping, it likely removed genes with few exons for which a 5′ exon was removed during splicing. Plots of DEU trends demonstrative of spurious transcription or alternative TSS are available in the GitHub repository https://github.com/snbogan/Sp_RRBS_ATAC.

Mann–Whitney *U* tests used to test for GO enrichment were parameterized with an alpha value of 0.05 and a minimum GO-term group size of 5 genes for gene-level enrichment. Alpha equaled 0.01 and the minimum GO-term group size equaled 25 genes for exon-level enrichment to account for a mean exon count of ~ 5 per gene in the *S. purpuratus* genome [120].

### DNA methylation analyses

RRBS sequences were trimmed and filtered with *TrimGalore* specifying the –rrbs option. Trimmed RRBS reads were mapped to the “Spur_3.1” genome assembly using *Bismark* [121] and methylation was called using the bismark_methylation_extractor command with default settings and SNPs unmasked. This decision was made because no panel or database of known SNPs is available for *S. purpuratus* and the pooled, single end RNA-seq and RRBS data in our study are insufficient for SNP calling [122, 123]. As such, genetic variation is expected to have imposed random error in our estimates of methylation level and differential methylation that reduce statistical power [124]. Coverage files were used for subsequent differential methylation analysis using an adapted *edgeR* workflow for RRBS data [125]. edgeR was selected for DM analysis to provide a statistical framework unified with estimations of DE and DEU such that methylation calls and RNA-seq counts underwent identical normalizations and were modeled using a negative binomial distribution fit with the same dispersion parameter. DM was tested among singular CpGs and genic features. To examine feature-specific responses by DNA methylation to environmental treatments, DM was estimated as logFC in percent methylation of all CpGs within the − 1 kb promoters, introns, and exons of a given gene or within singular exons and introns. Here, percent methylation was measured by *Bismark* from the proportion of methylated reads relative to all unmethylated plus methylated reads aligned to a CpG [125]. Methylation or DM within all introns/exons of a gene were used as a predictor in each reported model (e.g., models of baseline or differential gene expression) except for those predicting DEU. The DM of single exons was used as a predictor variable in models of DEU because this metric of splicing is estimated for singular exons. Counts summed across features were filtered to include genes represented by ≥ 10 reads across all samples. CpGs were also filtered to remove any loci aligned to fewer than 10 reads. Significant DM for both CpGs and genic features was assessed using an alpha value of FDR < 0.05. Functional enrichment of GO terms among differentially methylated genes was assessed using Mann–Whitney *U* tests input with signed, -log *p*-values using rank-based gene ontology analysis [120].

### Modeling gene regulation as a function of epigenomic variation

A Bayesian model fitting and selection workflow was used to quantitatively test the hypothesis that associations between differential methylation and gene regulation induced by maternal environment were influenced by chromatin accessibility. This workflow fit multiple models of DE logFC and DEU values that were composed of different combinations of singular and interacting predictors. These predictors related to DM across genic features, chromatin accessibility across genic features, and genic architecture. The most likely model was then selected using marginal likelihoods. The explanatory power of significant parameters within the selected model was then measured using a model averaging approach by which the likelihood of observed DE or DEU was estimated across models including a predictor relative to their likelihood under models lacking that predictor. This workflow enabled objective effect estimation regarding how predictor variables should behave or interact and reduced spurious correlation, reduced false positive predictions, and promoted reproducibility.

Sixteen iterative models were fit to DE and DEU outcome variables each using weakly informative priors that assumed null effects of DM and its interactions on gene regulation. This null effect was assumed because of the frequent observation that DM does not correlate with plastic changes in gene expression. Model selection was used to compare each of the simple or complex iterative models and select the most likely iteration using Bayes factors, which compare marginal likelihoods, the likelihood of a model integrating over its parameter space, between models. Simply put, Bayes factors compare the relative likelihoods of models given the observed data. Bayes factors also reduce prediction error relative to other selection metrics [58]. A given parameter from the selected model was determined to have a significant effect if it passed a probability of direction test, a Bayesian corollary of the *p*-value, by which 95% of an effect’s posterior probability must fall above or below 0. To avoid false positive predictions, significant effects were also evaluated using an inclusion Bayes factor, which averages the likelihood of observed outcome variables given all models that include a parameter and divides that value by the averaged likelihoods across all models that exclude it [126]. An inclusion Bayes factor ≥ 3 was considered evidence of significant explanatory power. We have reported effects as significant if they passed probability of direction and inclusion Bayes factor tests.

Studentized, gaussian linear models were fitted to measures of DE and DEU as functions of DM in − 1 kb promoters, introns, and/or exons, as well as logCPM, chromatin accessibility across genic features, and components of genic architecture such as the total length of genic features. Models of DEU also included predictors related to singular exons at which DEU was measured: DM of the corresponding exon, exon number, and exon length. All models were fitted using the R package *brms* v2.14.0, an R interface to the Stan programming language for specifying Bayesian models [127]. Models of DE and DEU were specified with weakly informative normal priors (mean = 0; SD = 0.5) for both slope (*β*) and intercept parameters. Posterior distributions were sampled using 4 chains at 20,000 iterations each, including 5000 warmup iterations.

Models were fitted with scaled Z-score transformations of continuous variables in order to improve model convergence and allow for comparisons of posterior *β* parameter distributions for predictors of different dependent variables. Unlike models of plastic changes in gene regulation (DE and DEU), models of baseline expression (logCPM) and the occurrence of transcript variants did not undergo model and parameter selection. logCPM was measured using a gaussian linear model. The binomial presence/absence of transcript variants per gene was measured using a generalized linear model set to a Bernoulli distribution. Both models were assigned weakly informative priors for all parameters (intercepts and *β* = 0 ± 0.5). Linear models of DE and DEU were fit with studentized model families to reduce prediction of artificially high or low outcome variables. To account for variation in RRBS read coverage across the data, the selected model was then refit to include an error parameter for estimated methylation and differential methylation that equaled the inverse CpG coverage of each gene or feature in the dataset. RRBS CpG coverage per feature is described in Additional file 1 for reported models (Additional file 1: Figs. S12 and S18). Posterior predictive checks were used to evaluate selected model predictions according to observed data (Additional file 1: Figs. S10 and S16).

Model selection was performed using the “bayesfactor_models” function in *bayestestR* v0.9.0 [128] to compare the likelihoods of models fit with iterative combinations of predictor variables. Probability of direction tests were conducted using the “posterior_interval” function of *brms* [127]. Inclusion Bayes factors for parameters were measured across all iterative models using the “bayesfactor_inclusion” function of *bayestestR* v0.9.0 [128]. Additional validation was performed on selected models using leave one out cross-validation and posterior predictive checks. Information about validation and predictions of selected models are available in Additional file 1. The specifications and relative likelihoods of selected and unselected models are available in the following GitHub repository: https://github.com/snbogan/Sp_RRBS_ATAC.

## Supplementary Information

**Additional file 1:** **Tables S1–S2.** Summaries of posterior probabilities for parameters from differential expression and differential exon use models. **Figs. S1–S6.** Plots of ATAC-seq quality checks and relationships among baseline or differential accessibility, DNA methylation, and gene expression. **Fig. S7–S12.** Figures plotting quality checks of differential expression model. **Fig. S13–S18.** Figures plotting quality checks of differential exon use model.

**Additional file 2.** File containing genewise parameter estimates for maternal and developmental effects on differential expression and differential exon use.

**Additional file 3.** File containing the results of Mann Whitney U and Fisher's exact tests for gene ontological enrichment among gene sets.

**Additional file 4.** File containing library quality and alignment metrics for RNA-seq and RRBS replicates.

## Data Availability

All data generated or analyzed during this study are included in this published article, its supplementary information files, and publicly available repositories, which we describe below. Raw RNA-seq and RRBS fastq files associated with this study are available through the NCBI Short Read Archive under the accession PRJNA548926. Scripts associated with trimming, mapping, and counting RNA-seq reads and CpG methylation are available in the following GitHub repository: https://github.com/mariestrader/S.purp_RRBS_RNAseq_2019. Code and genomic data files (all data except for fastq or bed files available on SRA) corresponding to all analyses reported in this study, their intermediate files, and outputs can be found in the following GitHub repository: https://github.com/snbogan/Sp_RRBS_ATAC. This repository’s DOI is as follows: https://doi.org/10.5281/zenodo.7729770.

## Abbreviations

ASG: Differentially spliced gene
ATAC: Assay for transposase-accessible chromatin
BP: Base pairs
CDS: Coding domain sequence
CPM: Counts per million
DE: Differential expression
DEG: Differentially expressed gene
DEU: Differential exon use
FC: Fold change
GBM: Gene body methylation
GLM: Generalized linear model
GO: Gene ontology
HPF: Hours post-fertilization
MF: Molecular function
RRBS: Reduced representation bisulfite sequencing
SNP: Single-nucleotide polymorphism
TGP: Transgenerational plasticity
TSS: Transcriptional start site
