# Supplementary material for: Associations between DNA methylation and gene regulation depend on chromatin accessibility during transgenerational plasticity
Source: BMC Biol. 2023 Jun 26;21:149. doi: 10.1186/s12915-023-01645-8 (PMC10294446; doi:10.1186/s12915-023-01645-8)
Supplement: Supplementary file 1 — Additional file 1: Tables S1–S2. Summaries of posterior probabilities for parameters from differential expression and differential exon use models. Figs. S1–S6. Plots of ATAC-seq quality checks and relationships among baseline or differential accessibility, DNA methylation, and gene expression. Fig. S7–S12. Figures plotting quality checks of differential expression model. Fig. S13–S18. Figures plotting quality checks of differential exon use model. [file 12915_2023_1645_MOESM1_ESM.pdf]

Supplemental Results:

Associations between DNA methylation and gene regulation depend on chromatin accessibility during transgenerational plasticity

Samuel N Bogan[1], Marie E Strader[1,2], Gretchen E Hofmann[1]

[1]Department of Ecology, Evolution and Marine Biology, University of California Santa Barbara

[2]Department of Biology, Texas A&M University

Corresponding Author:

Samuel N Bogan

snbogan@ucsb.edu

Table of Contents:

A. General supplemental figures (p. 3 – 8)

B. Specification and diagnostics for selected model of differential expression under maternal upwelling as a function of intron differential methylation (p. 9 – 15)

C. Specification and diagnostics for selected model of differential exon use under maternal upwelling as a function of differential exon methylation (p. 16 – 22)

General supplemental figures

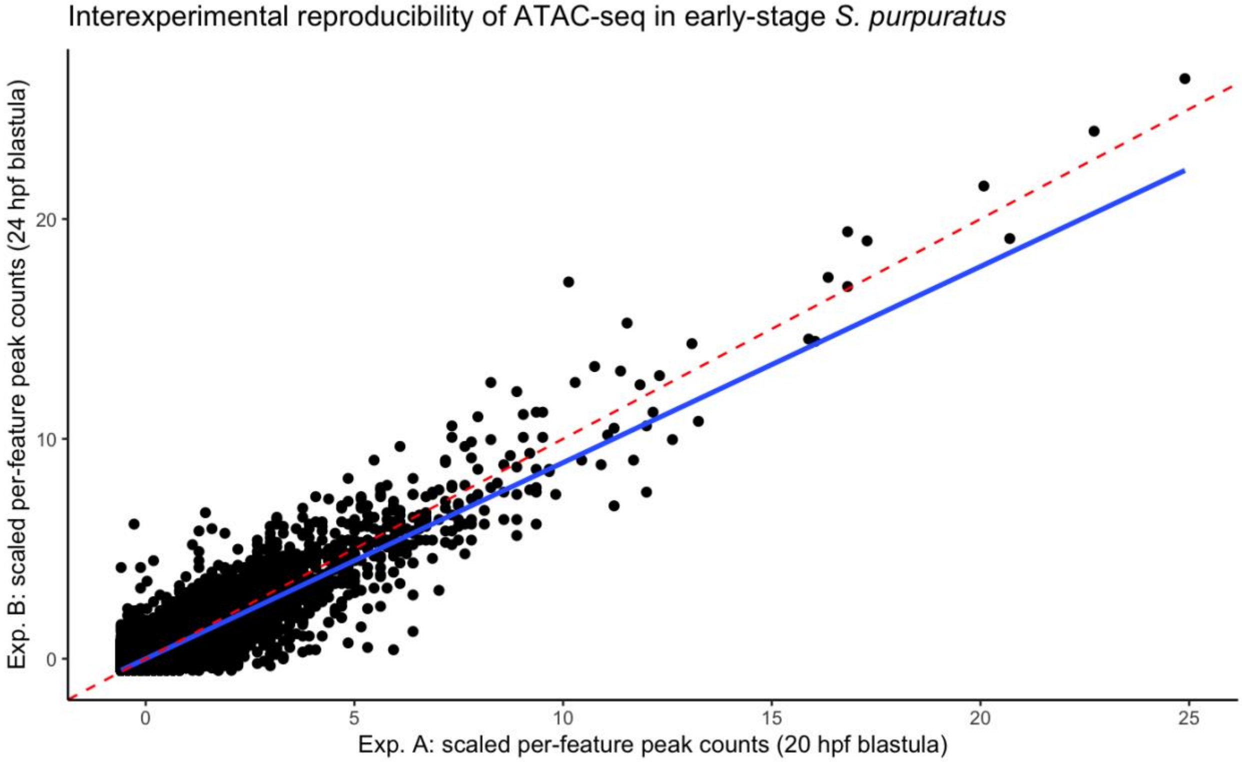

Figure S1: Between-experiment correlations in ATAC-seq estimates of chromatin accessibility across genomic features. Points represent mean-standardized, genewise estimates of chromatin accessibility peaks as measured by ATAC-seq at TSS, exons, and introns. The two axes depict peak counts estimated in two different experiments. The x-axis depicts data from GEO omnibus accession GSE160461 collected from 20 hpf *Strongylocentrotus purpuratus* blastula. The y-axis depicts data from the GEO omnibus accession GSE96927 collected from 24 hpf *S. purpuratus* blastula. The blue, solid line depicts the fitted linear regression (slope = 0.89; $R^2 = 0.80$; $p < 0.0001$). The red, dashed line represents a slope of 1.0. n = 2 replicate ATAC-seq libraries for experiment A; n = 4 replicate ATAC-seq libraries for experiment B.

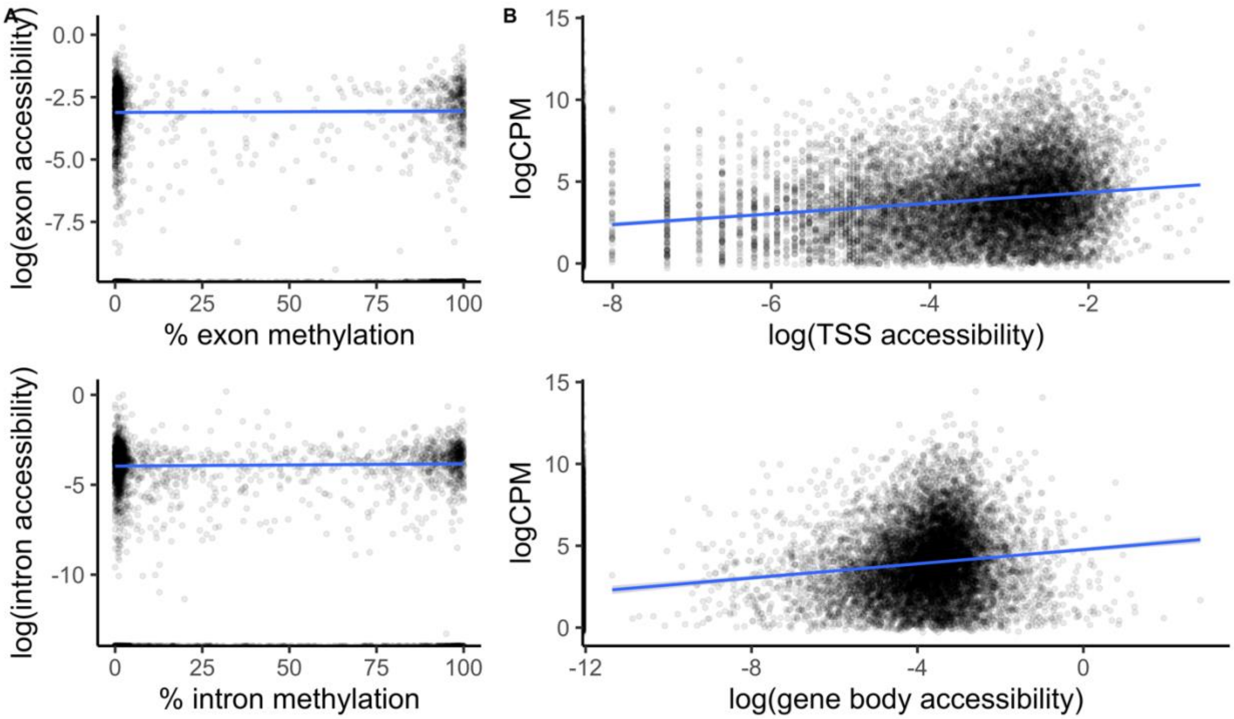

Figure S2: Relationships between baseline gene body methylation, chromatin accessibility, and gene expression. (A) % CpG methylation and log chromatin accessibility of exons (top) and introns (bottom) are plotted against one another. (B) TSS accessibility (top) and gene body accessibility (bottom) are plotted against transcript abundance measured as logCPM. n = 12 RNA-seq and RRBS replicate libraries; n = 3 ATAC-seq replicate libraries.

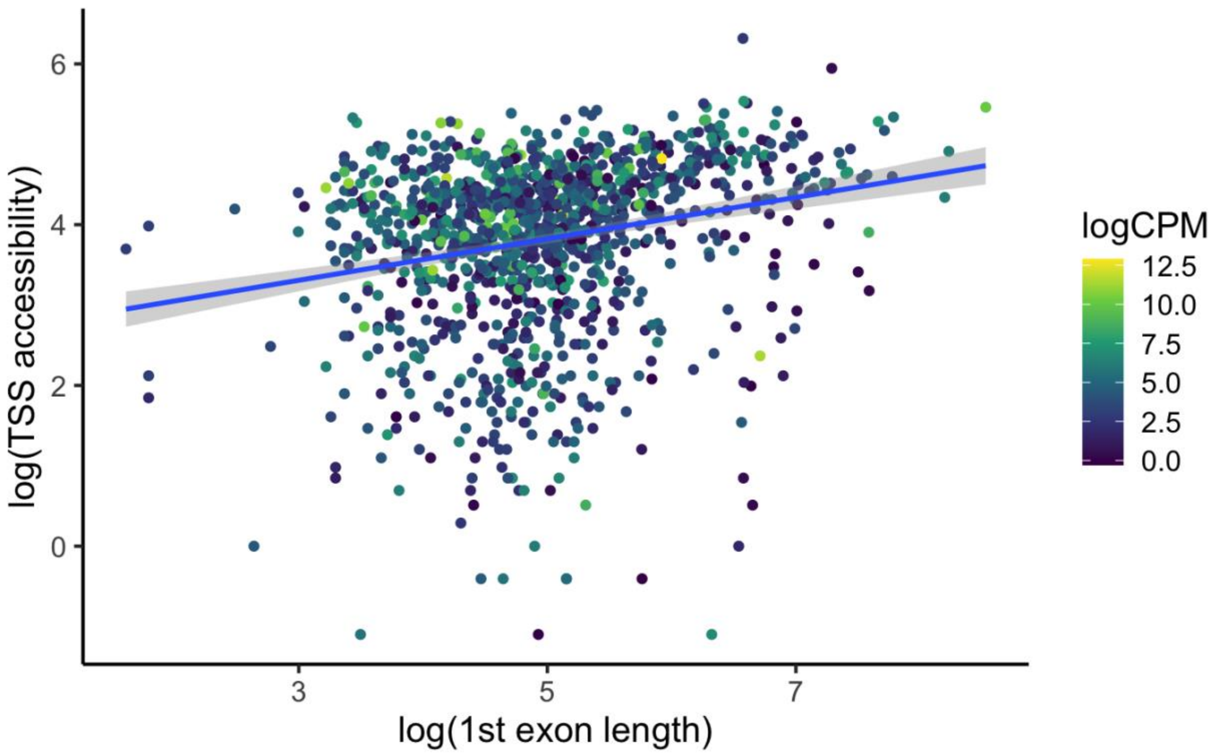

Figure S3: Positive correlations between log_{10} TSS accessibility, 1st exon length, and baseline gene expression (logCPM). n = 3 ATAC-seq replicate libraries.

General supplemental figures

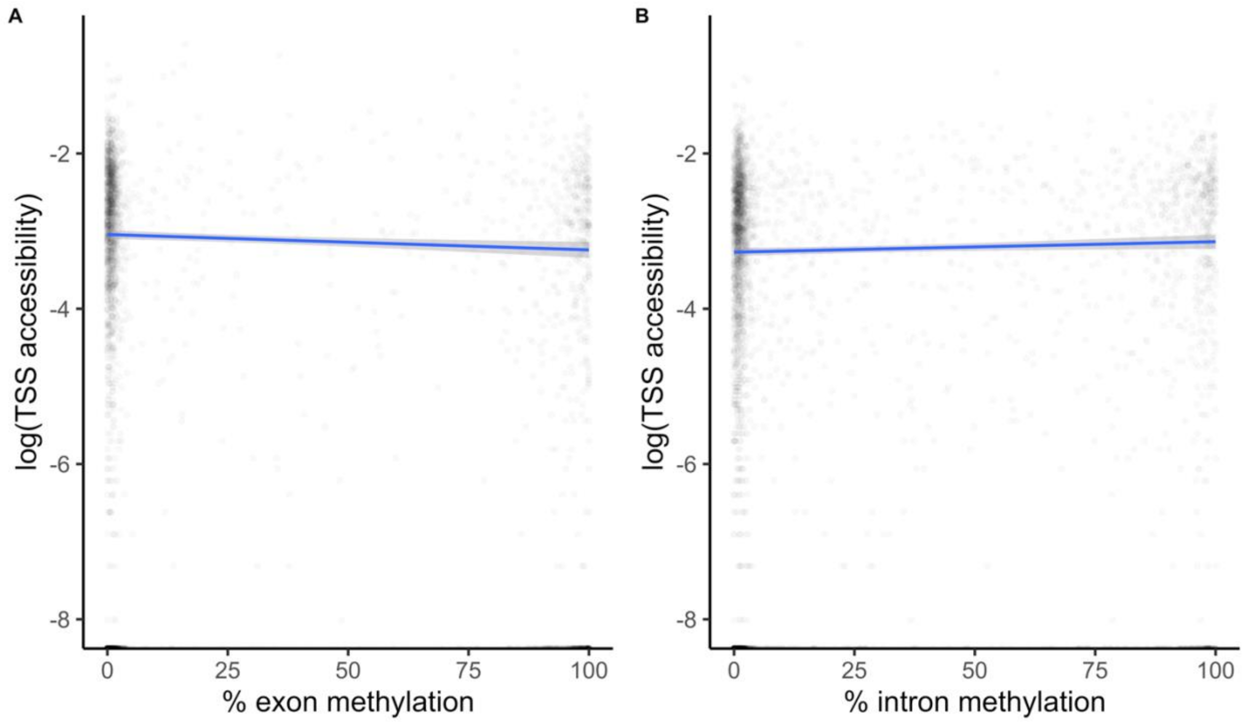

Figure S4: Relationships between log_{10} TSS accessibility and gene body methylation at (a) exons and (b) introns. n = 12 RRBS replicate libraries; n = 3 ATAC-seq replicate libraries.

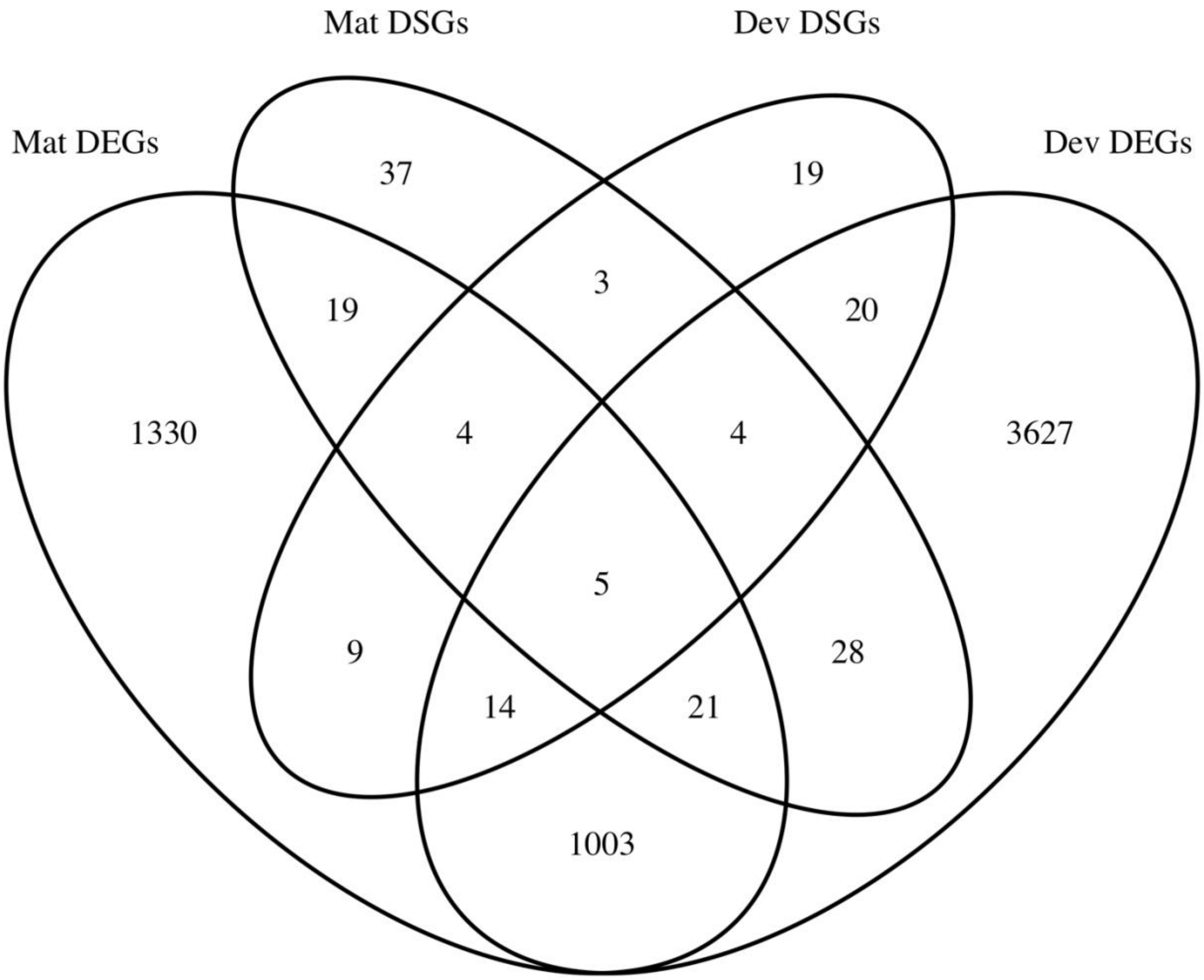

Figure S5: Overlap among differentially expressed (DEGs) and differentially spliced genes (DSGs) under maternal and developmental exposure to upwelling (FDR < 0.05). n = 12 RNA-seq replicate libraries.

General supplemental figures

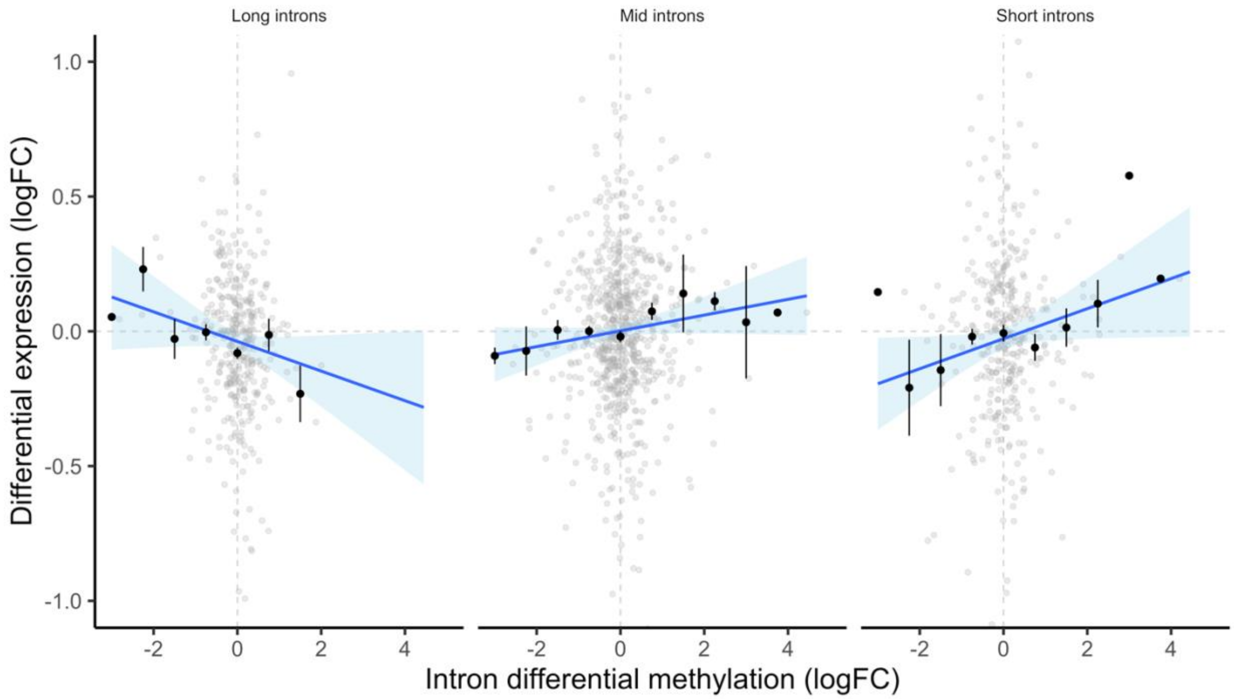

Figure S6: Differential intron methylation affected expression conditional upon intron length. Differential gene expression under maternal upwelling is plotted against mean intron differential methylation. Data are grouped based on genic intron length quartiles. 'Long introns' and 'short introns' denote highest and lowest quantiles. Linear regressions are fitted across observed values. Average logFC across binned intron differential methylation is plotted as black points ± SE. n = 12 RNA-seq and RRBS replicate libraries.

Specification and diagnostics for selected model of differential expression under maternal upwelling as a function of intron differential methylation

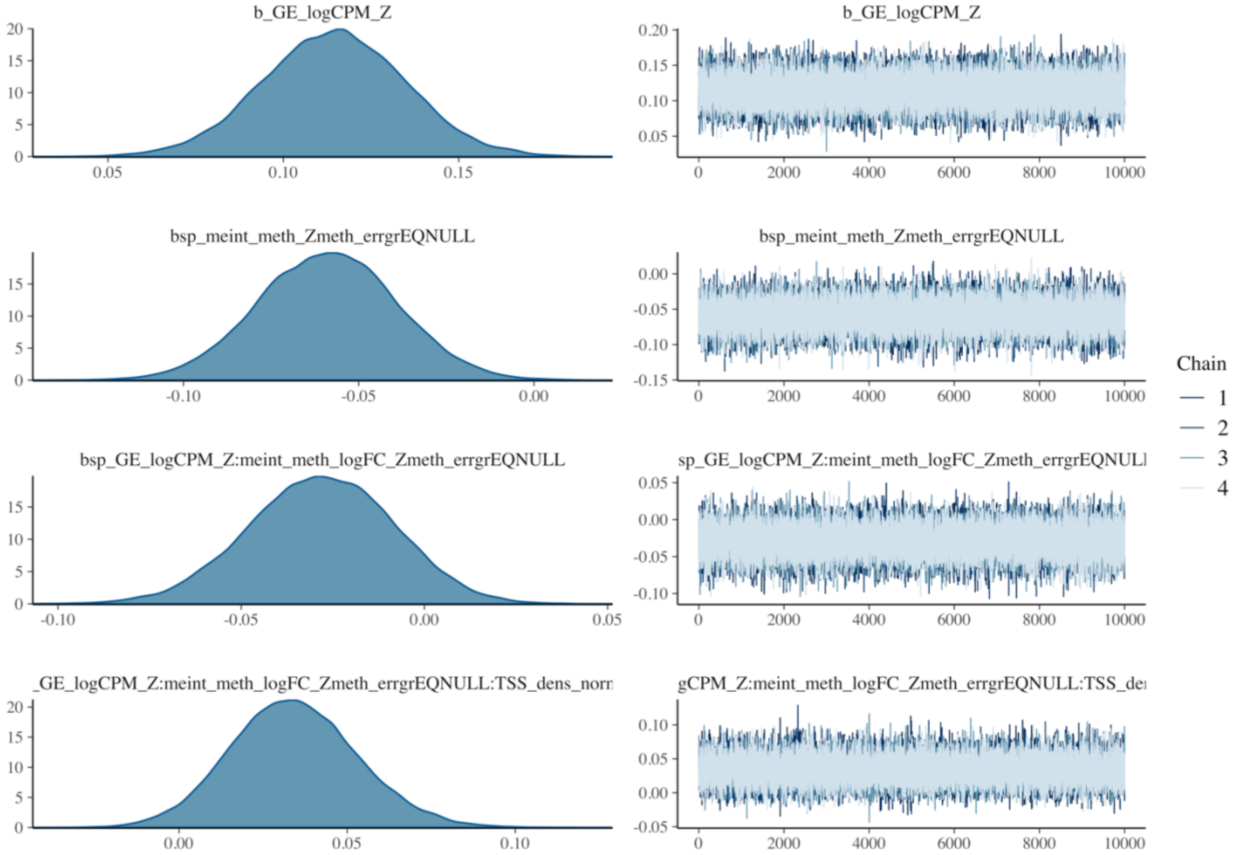

Figure S7: Posterior distributions and MCMC chains for β parameters of the selected model predicting differential expression under maternal upwelling as a function of differential intron methylation. "_*Z*" is appended to the end of parameters that were scaled to *Z*-scores during model fitting in order to improve run time and convergence of MCMC chains. 'GE_logCPM' denotes logCPM of gene expression. 'int_meth' denotes baseline % methylation of intronic CpGs. 'int_meth_logFC' represents log_2FC of differential intron methylation across genes. 'TSS_dens_norm' represents the density of chromatin accessibility at ± 500 bp TSS regions. n = 12 RNA-seq and RRBS replicate libraries; n = 3 ATAC-seq replicate libraries.

Specification and diagnostics for selected model of differential expression under maternal upwelling as a function of intron differential methylation

Parameter	5% interval	95% interval
b_Intercept	-4.156556e-02	2.395851e-02
b_GE_logCPM_Z*	8.061346e-02	1.477246e-01
b_int_meth_Z*	-9.143179e-02	-2.654733e-02
b_GE_logCPM_Z:int_meth_logFC_Z	-6.138834e-02	3.737507e-03
b_GE_logCPM_Z:int_meth_logFC_Z:TSS_dens_norm_Z*	4.109852e-03	6.619737e-02
sigma	5.642531e-01	6.325475e-01
nu	2.543551e+00	3.385978e+00
Intercept	-4.156556e-02	2.395851e-02

Table S1: Posterior intervals representing probability of direction tests applied to selected model of differential expression under maternal upwelling. Significant effects have posterior probabilities for which >95% of the distribution falls above or below 0. "_Z" is appended to the end of parameters that were scaled to Z-scores during model fitting in order to improve run time and convergence of MCMC chains. 'GE_logCPM' denotes logCPM of gene expression. 'int_meth' denotes baseline % methylation of intronic CpGs. 'int_meth_logFC' represents log_2FC of differential intron methylation across genes. 'TSS_dens_norm' is the density of chromatin accessibility at ± 500 bp TSS regions. An asterisk denotes methylation parameters modeled with an error term equaling inverse gene-level intron CpG coverage per gene. Asterisks denote significant fixed effects as evidence by probability of direction. n = 12 RNA-seq and RRBS replicate libraries; n = 3 ATAC-seq replicate libraries.

Specification and diagnostics for selected model of differential expression under maternal upwelling as a function of intron differential methylation

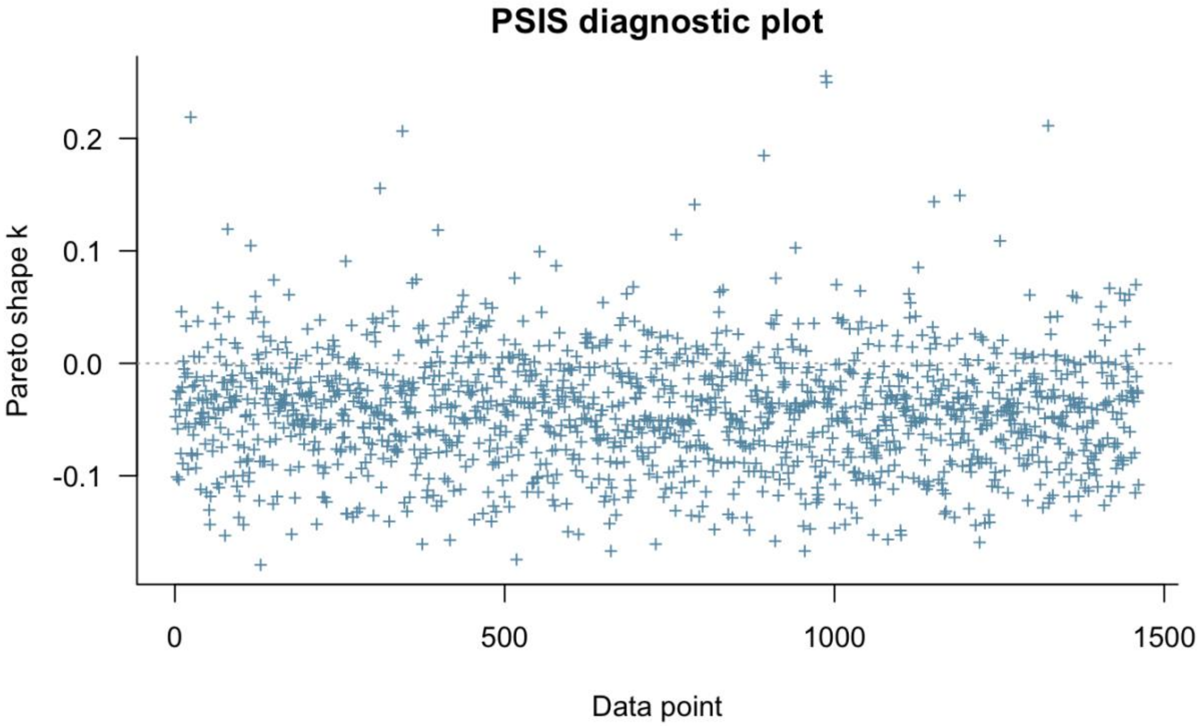

Figure S8: Leave-one-out estimates of leverage for observed data fit to selected model of differential expression under maternal upwelling as a function of differential intron methylation. Observations with pareto shape k > 0.4 are deemed to have moderate leverage capable of biasing model fitting. Observations with pareto shape k > 0.7 possess high leverage. n = 12 RNA-seq and RRBS replicate libraries; n = 3 ATAC-seq replicate libraries.

Specification and diagnostics for selected model of differential expression under maternal upwelling as a function of intron differential methylation

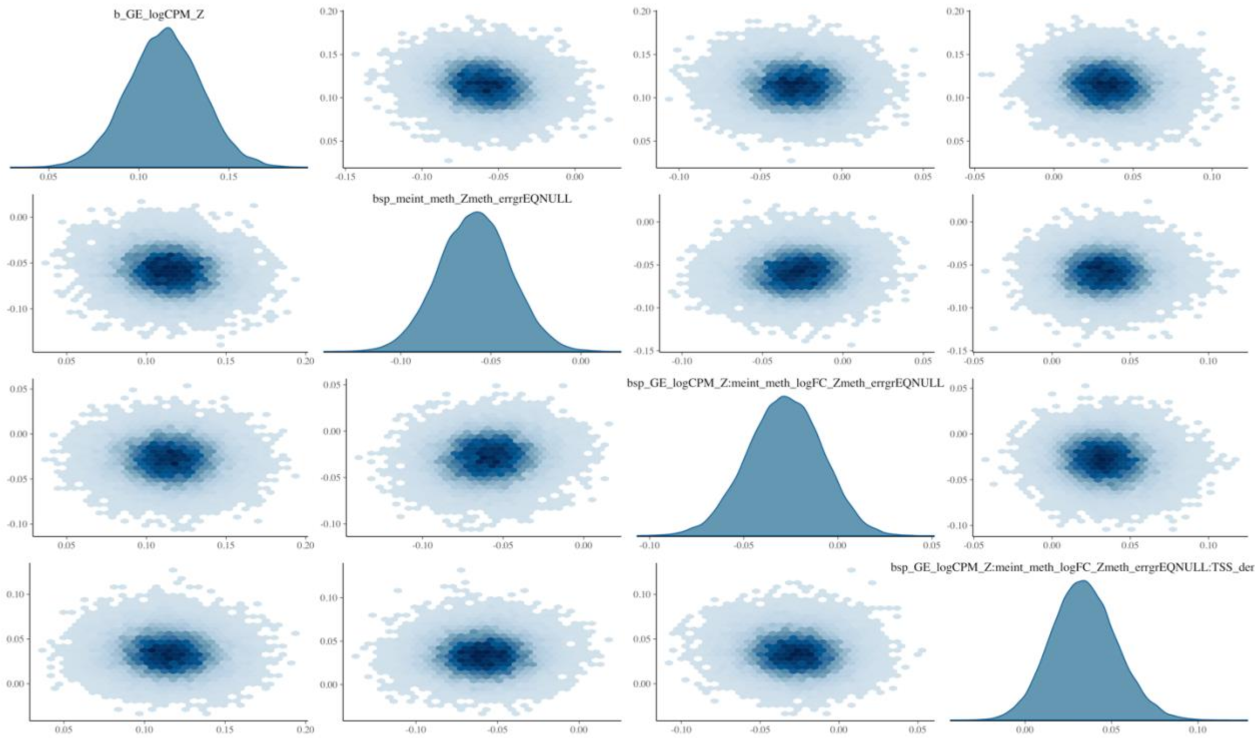

Figure S9: Correlation matrix of β posterior draws for fixed effects in selected model of differential expression under maternal upwelling as a function of differential intron methylation. Darker blue depicts greater point density. "_*Z*" is appended to the end of parameters that were scaled to Z-scores during model fitting. 'GE_logCPM' denotes logCPM of gene expression. 'int_meth' denotes baseline % methylation of intronic CpGs. 'int_meth_logFC' represents log_2FC of differential intron methylation across genes. 'TSS_dens_norm' depicts the density of chromatin accessibility at ± 500 bp TSS regions. n = 12 RNA-seq and RRBS replicate libraries; n = 3 ATAC-seq replicate libraries.

Specification and diagnostics for selected model of differential expression under maternal upwelling as a function of intron differential methylation

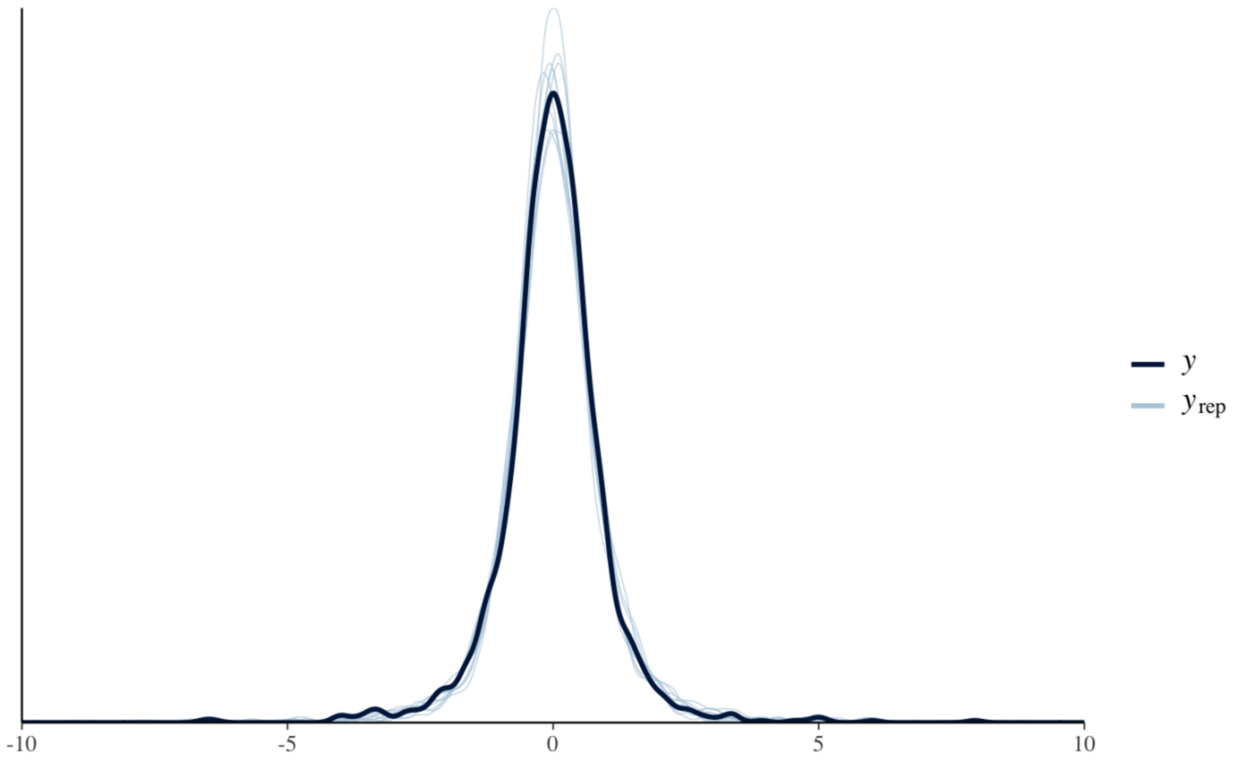

Figure S10: Posterior predictive check of selected model predicting differential expression under maternal upwelling as a function of differential intron methylation. The x-axis depicts Z score-scaled differential expression logFC values. The y axis the density distribution of observed and predicted logFC. The black line (γ) depicts the distribution of observed data. Blue lines (γ_{rep}) depict iterative distributions of model predictions. n = 12 RNA-seq and RRBS replicate libraries; n = 3 ATAC-seq replicate libraries.

Specification and diagnostics for selected model of differential expression under maternal upwelling as a function of intron differential methylation

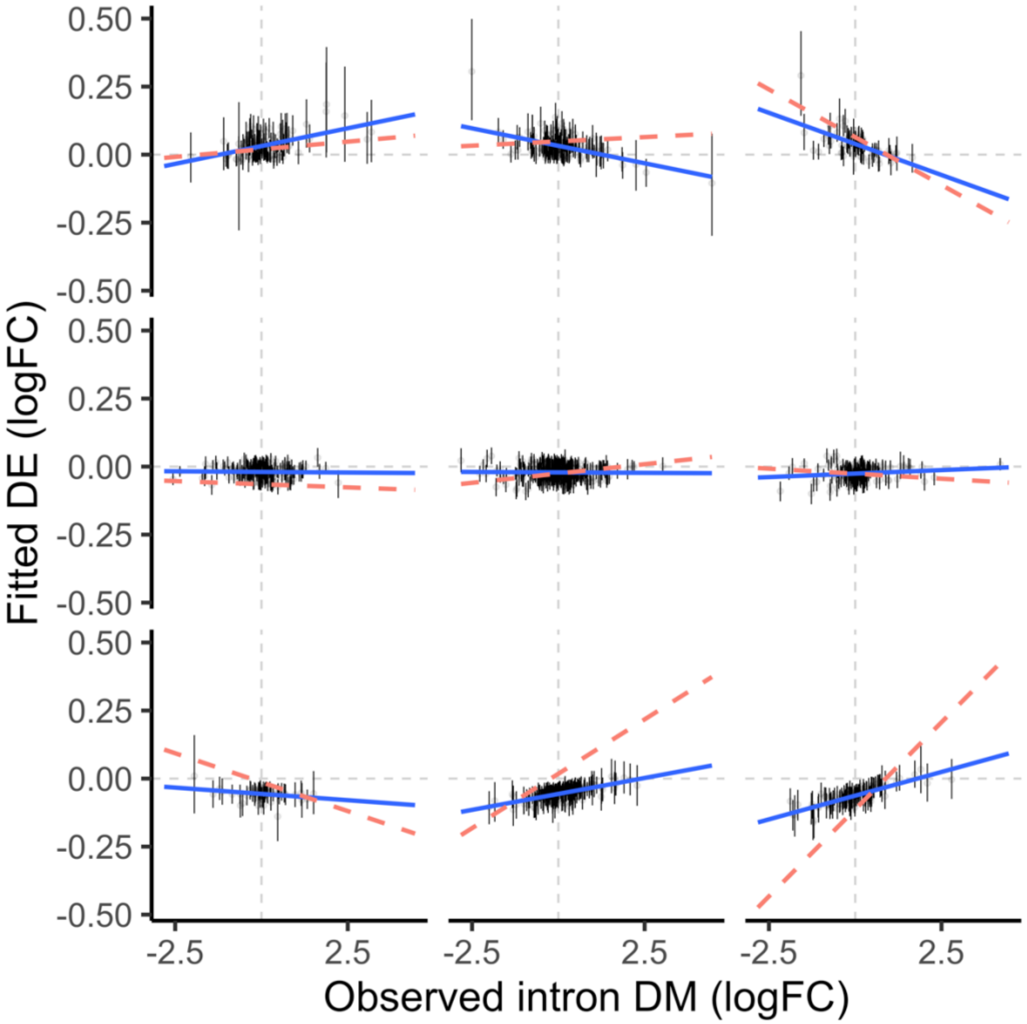

Figure S11: Predictions of differential expression (DE) under maternal upwelling by selected model relative to intron differential methylation (DM), TSS accessibility (columns), and gene expression level (rows). Individual points represent fitted values ± 95% credibility intervals. ‘Low’ and ‘high’ groupings of TSS accessibility and logCPM represent observations in the bottom and top quartiles of these variables. Blue solid lines depict fitted regressions to predicted DE. Red dashed lines depict unfitted regressions to observed DE. n = 12 RNA-seq and RRBS replicate libraries; n = 3 ATAC-seq replicate libraries.

Specification and diagnostics for selected model of differential expression under maternal upwelling as a function of intron differential methylation

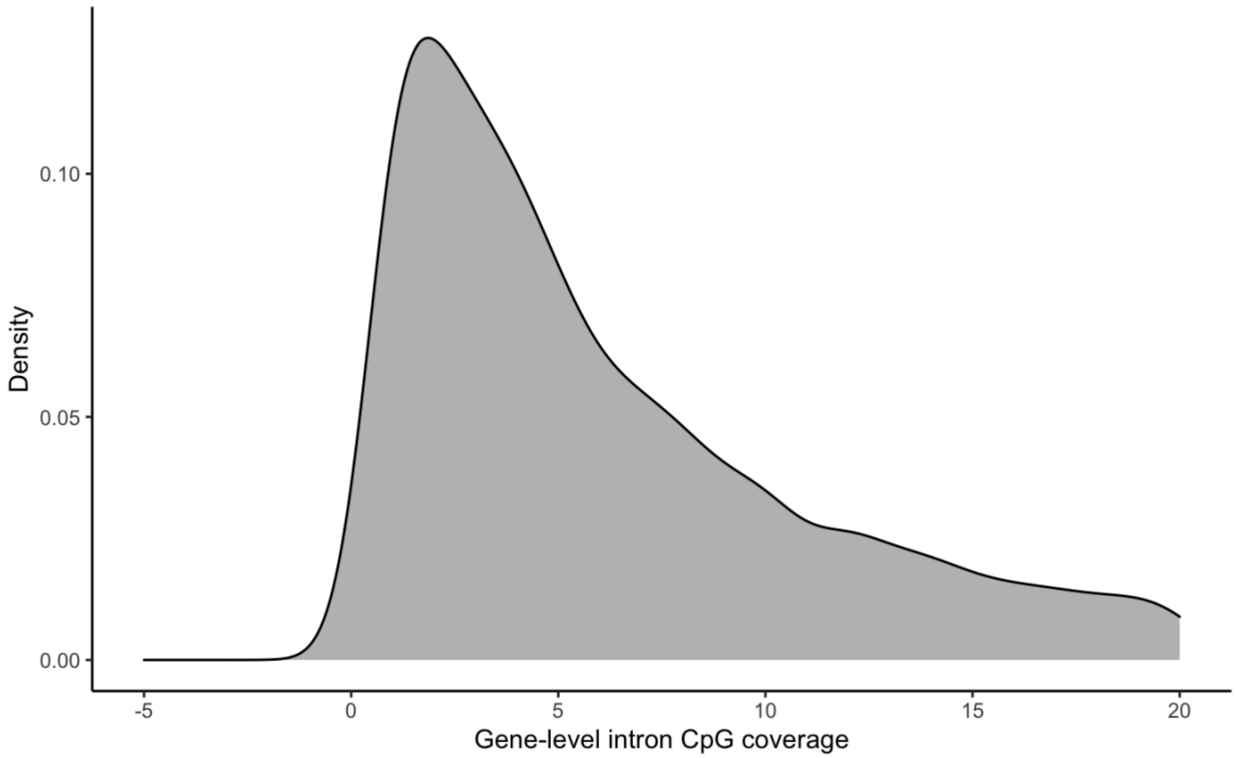

Figure S12: Gene-level RRBS CpG coverage of introns post-read count filtering. Mean coverage equaled 14.42 CpGs. Median coverage equaled 6 CpGs. n = 12 RRBS replicate libraries.

Specification and diagnostics for selected model for differential exon use under maternal upwelling as a function of differential exon methylation

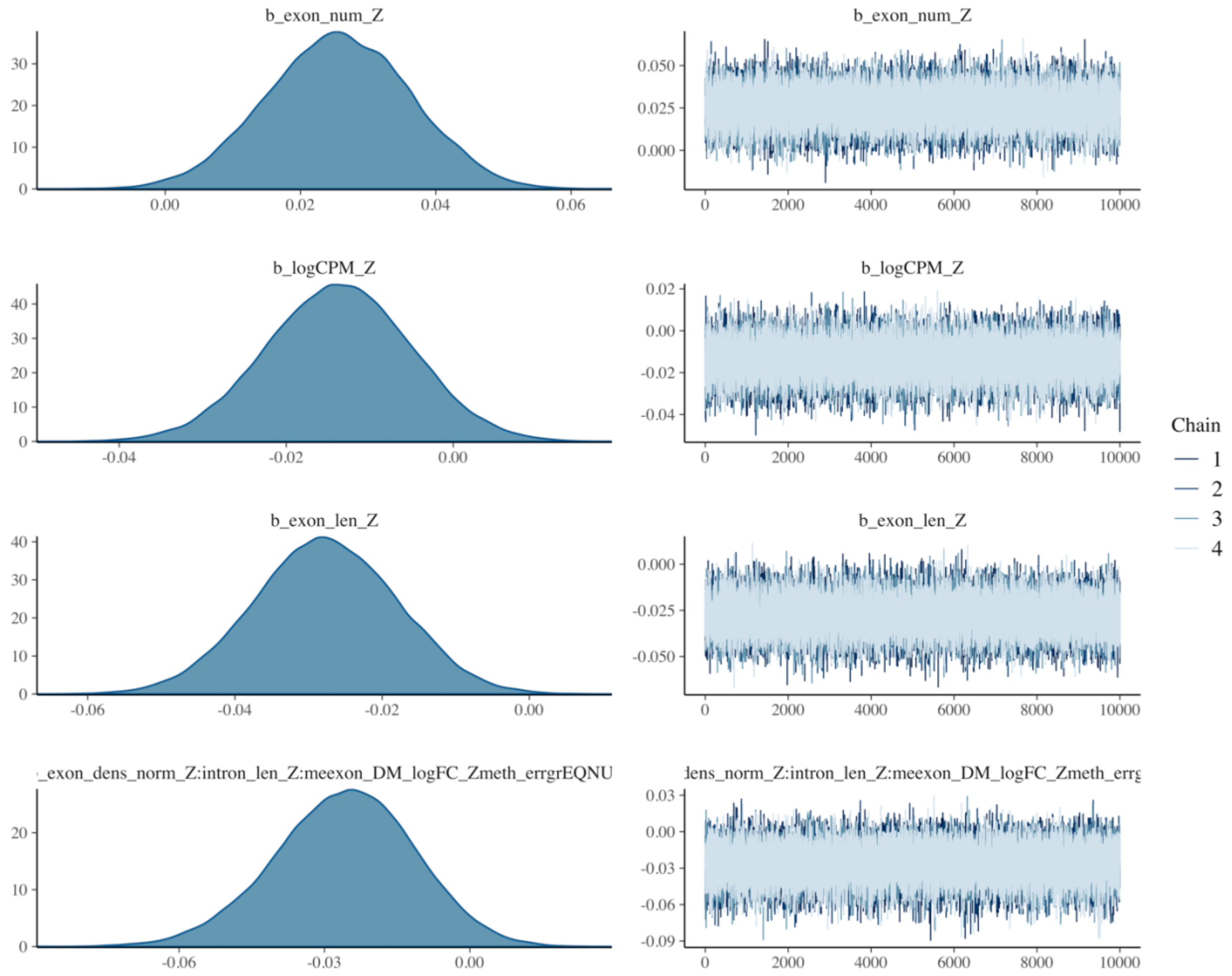

Figure S13: Posterior distributions and MCMC chains for β parameters of the selected model predicting differential exon use under maternal upwelling as a function of differential exon methylation. "_*Z*" is appended to the end of parameters that were scaled to *Z*-scores during model fitting in order to improve run time and convergence of MCMC chains. 'exon_num' represents exon number. 'logCPM' denotes logCPM of gene expression. 'exon_DM_logFC denotes $\log_2$FC values of exon differential methylation. 'intron_len' represents total genic intron length. 'exon_dens_norm' depicts the density of chromatin accessibility at across exons of the associated gene. n = 12 RNA-seq and RRBS replicate libraries; n = 3 ATAC-seq replicate libraries.

Specification and diagnostics for selected model for differential exon use under maternal upwelling as a function of differential exon methylation

Parameter	5% interval	95% interval
b_Intercept	-4.499084e-02	-1.880066e-02
b_exon_num_Z*	8.144410e-03	4.309011e-02
b_logCPM_Z	-2.821506e-02	5.262227e-04
b_exon_len_Z*	-4.378426e-02	-1.173342e-02
b_exoncoeffZ_exon_dens_norm_Z:intron_len_Z: exon_DM_logFC_Z*	-5.064898e-02	-2.771061e-03
sigma	2.420005e-01	2.773288e-01
nu	1.030224e+00	1.190071e+00
Intercept	-4.499084e-02	-1.880066e-02

Table S2: Posterior intervals representing probability of direction tests applied to selected model of differential exon use under maternal upwelling. Significant effects have posterior probabilities for which >95% of the distribution falls above or below 0. "_*Z*" is appended to the end of parameters that were scaled to Z-scores during model fitting in order to improve run time and convergence of MCMC chains. 'exon_num' represents exon number. 'logCPM' denotes logCPM of gene expression. 'exon_DM_logFC' represents log_2FC of differential methylation at single exons. 'exon_dens_norm' depicts the density of chromatin accessibility at all exons within a gene. An asterisk denotes methylation parameters modeled with an error term equaling inverse exon CpG coverage per gene. Asterisks denote significant fixed effects as evidence by probability of direction.

Specification and diagnostics for selected model for differential exon use under maternal upwelling as a function of differential exon methylation

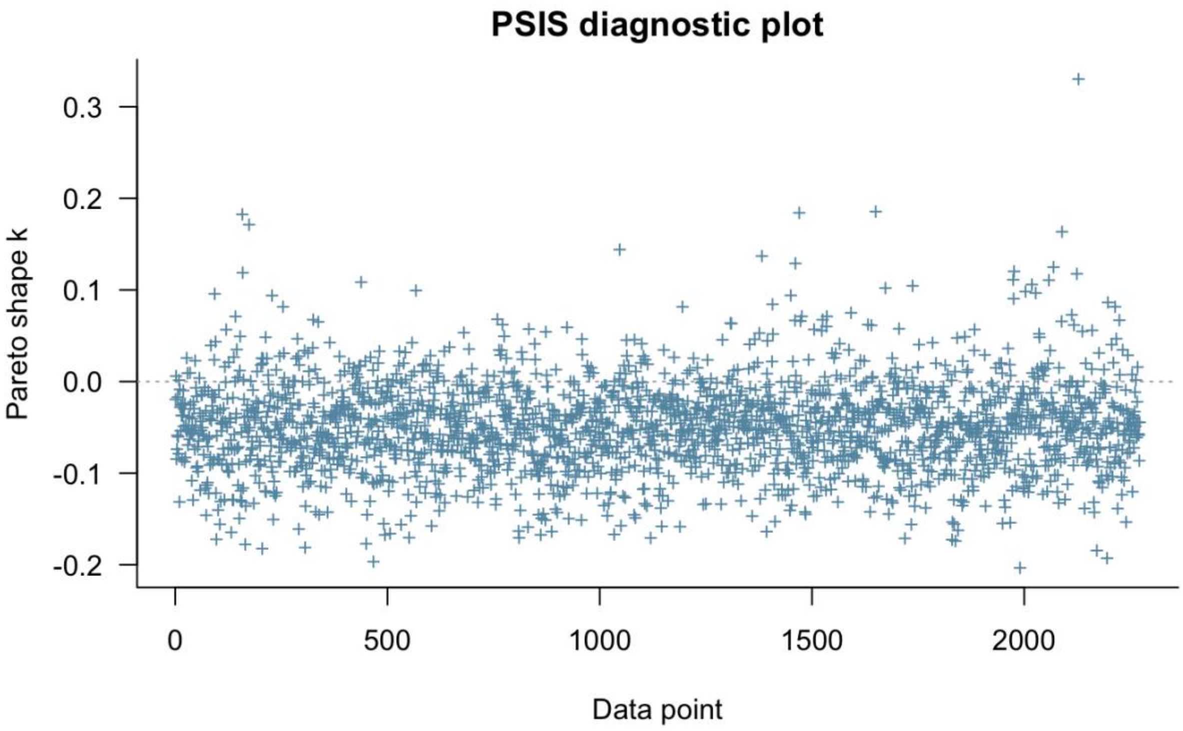

Figure S14: Leave-one-out estimates of leverage for observed data fit to selected model of differential exon use under maternal upwelling as a function of differential exon methylation. Observations with pareto shape k > 0.4 are deemed to have moderate leverage capable of biasing model fitting. Observations with pareto shape k > 0.7 possess high leverage. n = 12 RNA-seq and RRBS replicate libraries; n = 3 ATAC-seq replicate libraries.

Specification and diagnostics for selected model for differential exon use under maternal upwelling as a function of differential exon methylation

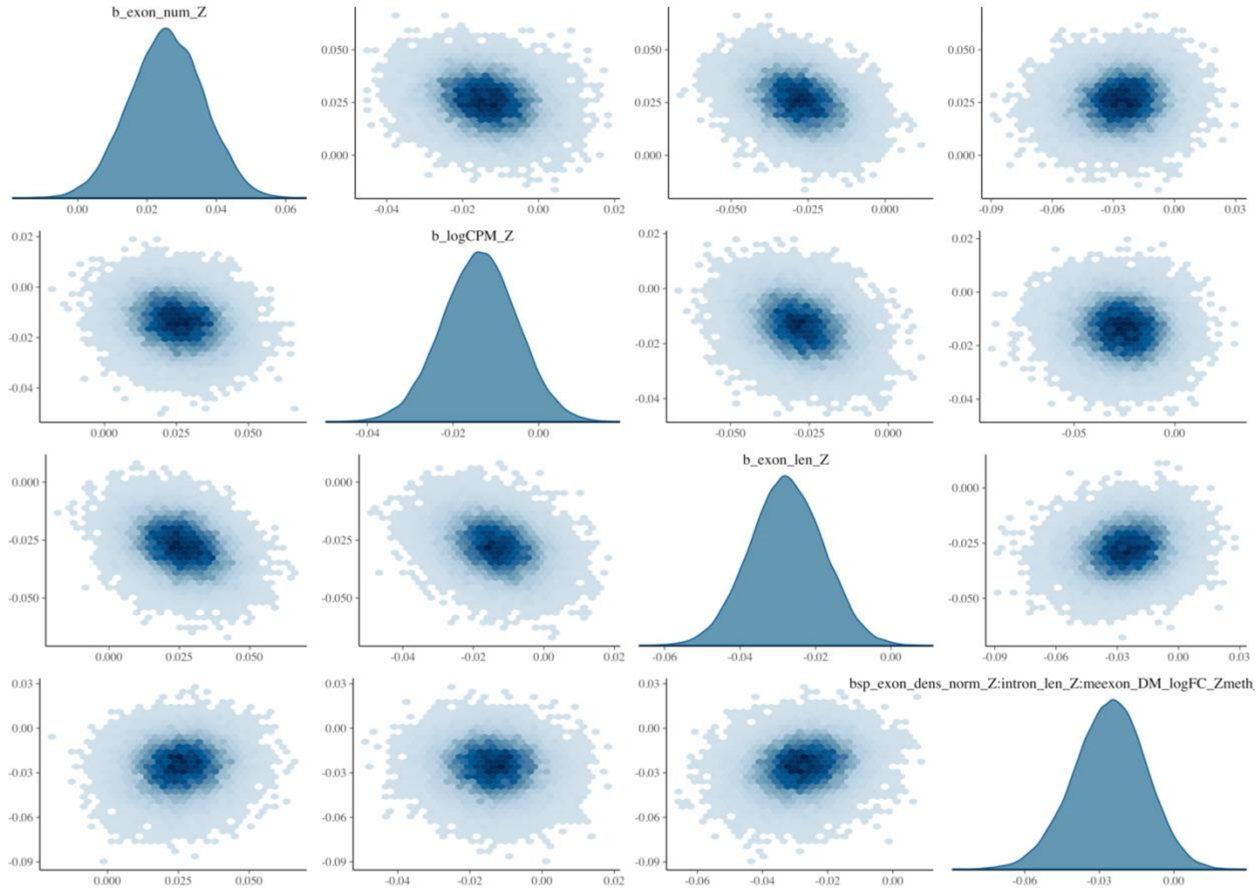

Figure S15: Correlation matrix of β posterior draws for fixed effects in selected model of differential exon use under maternal upwelling as a function of differential exon methylation. Darker blue depicts greater point density. "_Z" is appended to the end of parameters scaled to *Z*-scores during model fitting. 'exon_num' represents exon number. 'logCPM' denotes logCPM of gene expression. 'exon_DM_logFC' represents log_2FC of differential exon methylation across genes. 'exon_dens_norm' depicts the density of chromatin accessibility at exons within a gene. "intron_len" represents total intron length of a gene. n = 12 RNA-seq and RRBS replicate libraries; n = 3 ATAC-seq replicate libraries.

Specification and diagnostics for selected model for differential exon use under maternal upwelling as a function of differential exon methylation

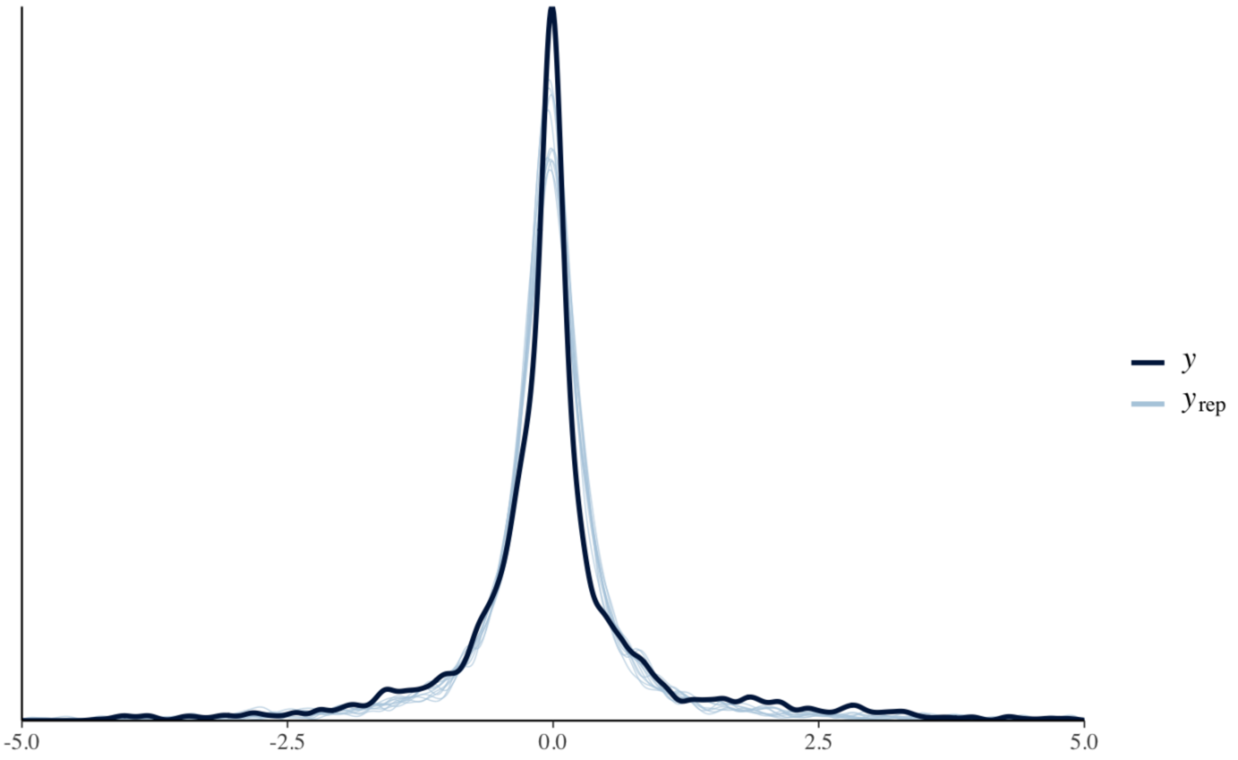

Figure S16: Posterior predictive check of selected model predicting differential exon use under maternal upwelling as a function of differential exon methylation. The x-axis depicts Z score-scaled differential exon use ΔlogFC values. The y axis the density distribution of obersved and predicted ΔlogFC. The black line (γ) depicts the distribution of observed data. Blue lines (γ_{rep}) depict iterative distributions of model predictions. n = 12 RNA-seq and RRBS replicate libraries; n = 3 ATAC-seq replicate libraries.

Specification and diagnostics for selected model for differential exon use under maternal upwelling as a function of differential exon methylation

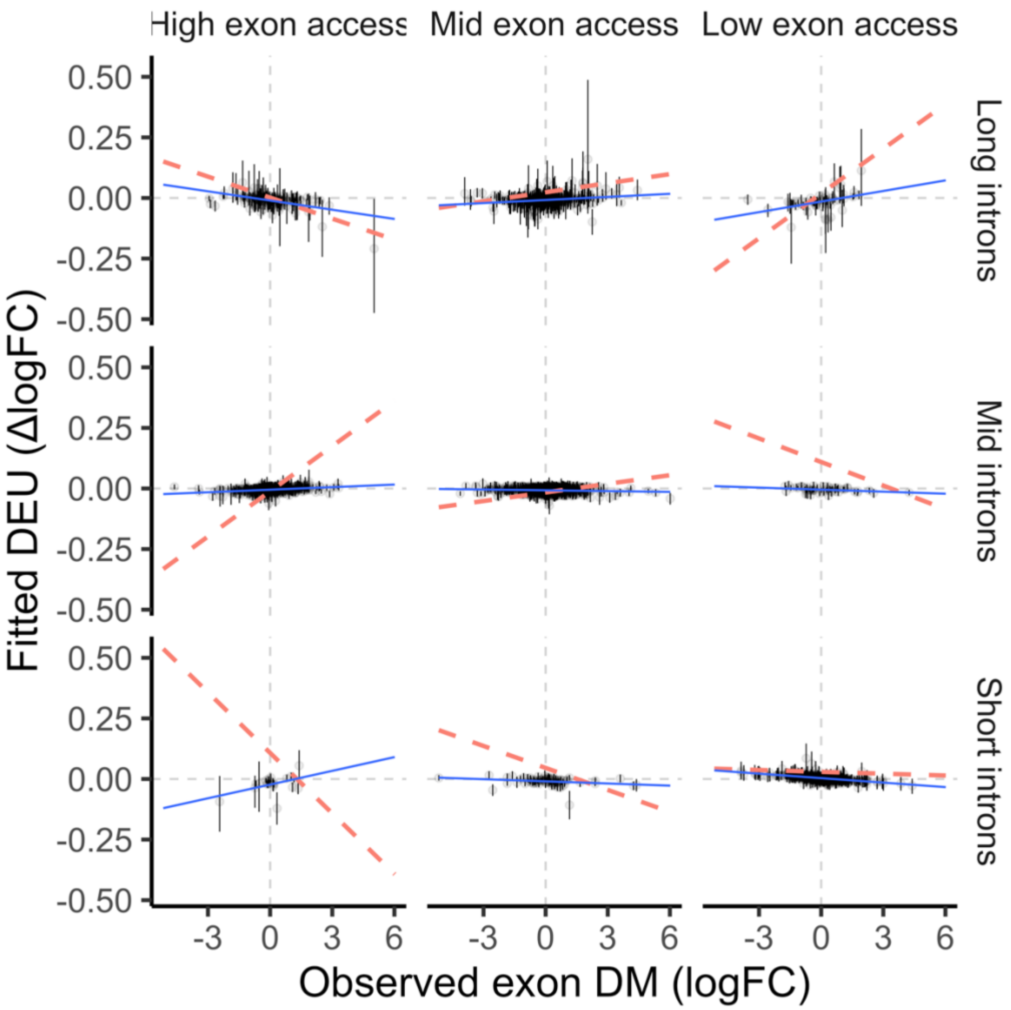

Figure S17: Predictions of differential exon use (DEU) under maternal upwelling by selected model relative to exon differential methylation (DM), exon accessibility (columns), and total genic intron length (rows). Individual points depict fitted values per exon ± 95% credibility intervals. 'Low' and 'high' groupings of exon accessibility and intron length represent observations in the bottom and top quartiles of these variables. Blue solid lines depict fitted regressions to predicted DEU. Red dashed lines depict unfitted regressions to observed DEU. n = 12 RNA-seq and RRBS replicate libraries; n = 3 ATAC-seq replicate libraries.

Specification and diagnostics for selected model for differential exon use under maternal upwelling as a function of differential exon methylation

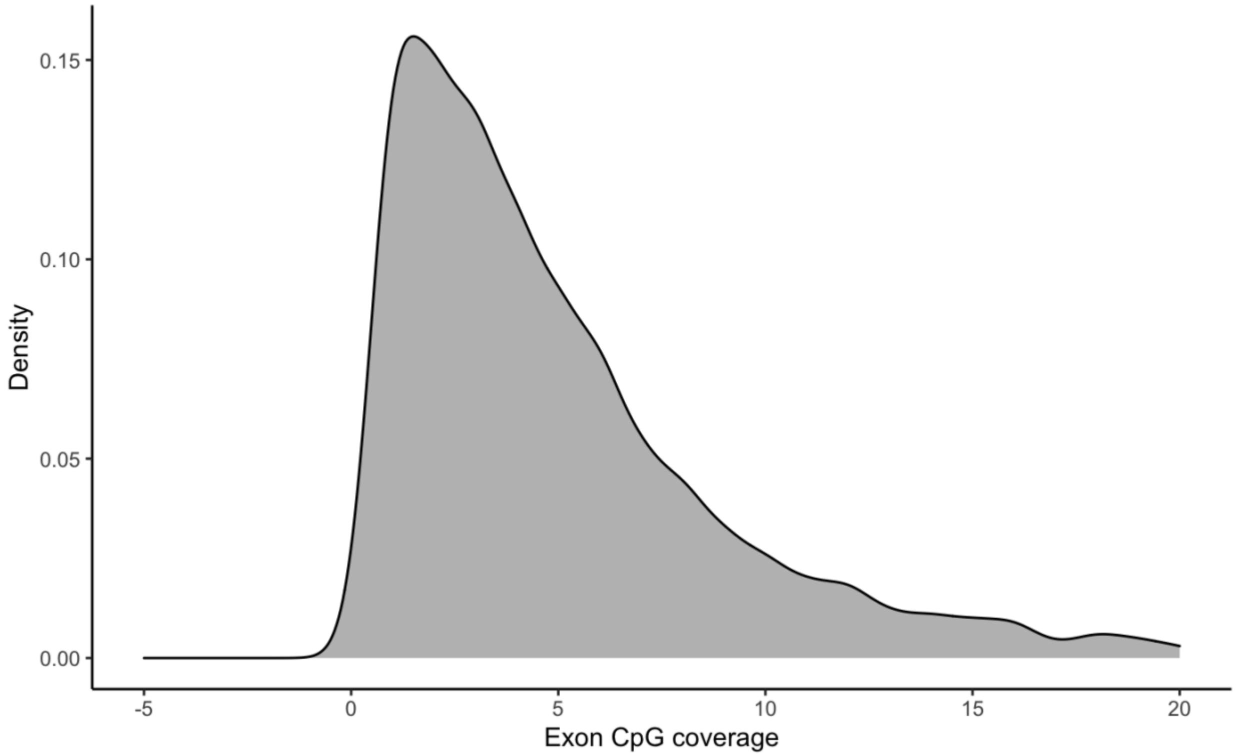

Figure S18: RRBS CpG coverage of individual exons post-read count filtering. Mean coverage equaled 5.42 CpGs. Median coverage equaled 4 CpGs. n = 12 RRBS replicate libraries.
